# Using SHAP and LIME to Explain Machine Learning Models Predicting Comorbid Depression and Stroke From Daily Dietary Nutrient Intake in a US Population‐Based Study

**DOI:** 10.1002/fsn3.71401

**Published:** 2025-12-30

**Authors:** Hongwei Liu, Minghui Wu, Peng Wei, Haixia Fan, Miaomiao Hou

**Affiliations:** ^1^ Department of Neurology, Taiyuan City Central Hospital The Ninth Clinical Medical College of Shanxi Medical University Taiyuan Shanxi Province China; ^2^ Department of Neurology Xuanwu Hospital of Capital Medical University Beijing China; ^3^ Department of Sleep Center First Hospital of Shanxi Medical University Taiyuan Shanxi Province China; ^4^ Department of Neurology, Shanxi Bethune Hospital, Shanxi Academy of Medical Sciences Third Hospital of Shanxi Medical University, Tongji Shanxi Hospital, Tongji Shanxi Hospital Taiyuan Shanxi Province China

**Keywords:** depression, dietary nutrient intake, machine learning models, NHANES, stroke

## Abstract

While comorbid depression and stroke are a major concern for public health, the effect of dietary nutrient patterns on their concurrent occurrence is still largely unexplored. From NHANES, a survey of the U.S. civilian, non‐institutionalized population, we included 814 participants with complete data on diet, depression, and stroke. Of these, 140 were identified with comorbid depression and stroke. Baseline characteristics were compared between groups, and Weighted Quantile Sum (WQS) regression was used to evaluate the collective effects of nutrient mixtures. Machine learning models aimed at predicting comorbid conditions were developed, incorporating Synthetic Minority Oversampling Technique (SMOTE) for oversampling and Boruta for selecting features. The interpretability of these models was analyzed using SHapley Additive exPlanations (SHAP) and Local Interpretable Model‐agnostic Explanations (LIME). Participants with comorbidities were younger and had lower socioeconomic status, along with reduced intake of thiamin, vitamin B6, total folate, added vitamin B12, and vitamin C. Although neither WQS‐negative nor WQS‐positive indices showed statistically significant associations with comorbidity risk, specific nutrients such as alcohol, alpha‐carotene, added vitamin B12, theobromine, and vitamin E emerged as predominant contributors within the mixture models. The Random Forest classifier achieved the highest area under the receiver operating characteristic curve (AUC = 0.945) when adjusted for covariates and maintained consistently high performance in the unadjusted setting. SHAP and LIME analyses consistently identified vitamin B1, vitamin B12, zinc, vitamin C, and caffeine as influential predictors, with SHAP plots revealing mirrored feature contribution patterns depending on comorbidity status. Covariate adjustment improved directional stability and interpretability, particularly in SHAP dependence plots and waterfall visualizations. LIME explanations at the individual level corroborated these findings, showing consistent yet class‐dependent feature effects. Although the overall mixture effect was not significant, machine learning identified nutrient‐specific signals associated with comorbid depression and stroke. These results indicate that integrating dietary indicators with explainable artificial intelligence may improve transparency in risk prediction and guide future longitudinal and interventional research.

## Graphical abstract

This study used interpretable machine learning to identify dietary nutrient patterns associated with depression‐stroke comorbidity in U.S. adults aged 50 years and older. While overall nutrient mixtures showed no significant association, specific micronutrients including vitamins B1, B12, and C, zinc, and caffeine consistently predicted comorbidity risk. The integration of SHAP and LIME improved interpretability, revealing nutrient‐specific influences and underscoring the potential of explainable AI for advancing nutritional precision medicine.

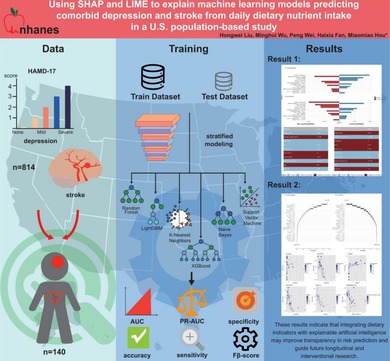

## 1 Introduction

Depression and stroke are two major health concerns worldwide, with evidence increasingly pointing to a mutual relationship that exacerbates disease progression and complicates treatment outcomes (Almeida 2023; Mu et al. 2024; Zhao et al. 2019). Epidemiological research indicates that stroke survivors are 2–3 times more prone to depression than the general population, and pre‐existing depression raises the likelihood of stroke by 30%–50% (Pan et al. 2011; Ashraf et al. 2024). This comorbidity is influenced by common pathophysiological mechanisms, including chronic inflammation, dysregulation of the hypothalamic–pituitary–adrenal axis, and endothelial dysfunction (Beurel et al. 2020; Leonard 2018; Wu, Zhang, et al. 2025) In clinical practice, pharmacological interventions are still the primary approach, but modifiable lifestyle factors, particularly the intake of dietary nutrients, have become key contributors to neurovascular health (Lobo et al. 2022; English et al. 2021; Spence 2019). For instance, lacking omega‐3 fatty acids, folate, and vitamin D is tied to higher risks of depression and stroke, while excessive intake of sodium and saturated fats might speed up damage to the brain's blood vessels (Spence 2019; Kris‐Etherton et al. 2021; Zielińska et al. 2022; Carson et al. 2020; Larsson et al. 2012). Yet, many existing studies rely on linear regression models, which are unable to adequately capture complex nutrient interactions or non‐linear dose–response relationships (White and Barnett 2019).

Machine learning (ML) presents a promising method for capturing high‐dimensional, non‐linear links between eating patterns and the comorbidity of depression and stroke (Reel et al. 2021; Miller et al. 2024; Libbrecht and Noble 2015). Compared to traditional statistical methods, ML applications in nutritional psychiatry have achieved higher predictive accuracy for mental health outcomes (Liu et al. 2022; Chekroud et al. 2016). Despite being a fundamental supervised ML approach, linear regression's potential is constrained by its use of linear transformations in the parameter space (Nguyen et al. 2025; Cherkassky and Ma 2009). Modern ML approaches extend these same linear‐algebra principles by incorporating matrix decomposition, vectorization, kernel‐based mappings, and related algebraic operations, allowing them to capture nonlinear relationships and intricate interaction structures among nutrient variables (Dwyer et al. 2018; Arfat et al. 2022; Ben‐Hur et al. 2008; Ching et al. 2018). These mechanisms allow contemporary ML models to capture higher‐order dependencies that traditional linear models cannot accommodate, offering a more flexible and informative framework for analyzing complex nutritional data (Eraslan et al. 2019; Zou et al. 2019). Nonetheless, a notable limitation continues: many ML models act as “black boxes,” giving limited clarity on the role of specific nutrients in risk predictions (Rudin 2019). This vagueness hampers clinical implementation, as clinicians require evidence they can interpret to endorse dietary recommendations (Holzinger et al. 2019). In response to this, techniques in explainable AI (XAI) such as SHapley Additive exPlanations (SHAP) and Local Interpretable Model‐agnostic Explanations (LIME) have been developed to evaluate feature importance and interpret model decisions at both global and local levels (Alkhanbouli et al. 2025; Ali et al. 2023). For example, SHAP values have been used to rank biomarkers in cardiovascular disease prediction, while LIME generates patient‐specific explanations for treatment responses (Haupt et al. 2025; Ladbury et al. 2022).

By applying SHAP and LIME approaches, this investigation seeks to clarify machine learning models that anticipate depression and stroke comorbidity based on daily nutrient intake. Our goal is to discover significant dietary elements and link predictive outcomes with clinical insights by merging population‐level feature importance (SHAP) with individual‐case explanations (LIME). Because both stroke and depression disproportionately affect adults in midlife and later life, with stroke incidence rising steeply after age 50 and depression risk peaking between 50 and 54 years, we focused our analysis on individuals aged ≥ 50 years (Xu et al. 2024; Feigin et al. 2025). By focusing on this age group, the study improved case representation and aligned with the population most affected by this comorbidity, thereby enhancing its significance in clinical and public health contexts. In doing so, our work contributes to the broader movement toward “nutritional precision medicine,” applying machine learning to inform tailored dietary interventions and guide strategies for reducing the burden of diet‐related neurovascular comorbidities.

## 2 Materials and Methods

### 2.1 Study Design and Study Population

**FIGURE 1:**
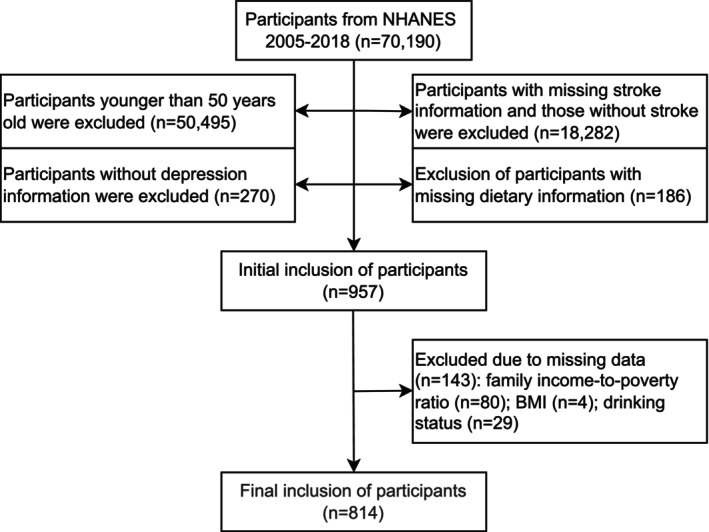
Flow chart of participant selection. BMI, body mass index; NHANES, National Health and Nutrition Examination Survey.

Data were obtained from the National Health and Nutrition Examination Survey (NHANES) cycles spanning 2005 to 2018. Out of the initial 70,190 participants, individuals below 50 years (*n* = 50,495) were excluded to target midlife and older age groups. Participants without stroke history information (*n* = 18,282) and depression status (*n* = 270) were additionally excluded. We further excluded individuals with missing data on key covariates, including family income‐to‐poverty ratio (*n* = 80), body mass index (BMI) (*n* = 34), and drinking status (*n* = 29). To guarantee accurate exposure assessment, participants who did not have dietary intake data were also excluded (*n* = 186). Finally, 814 participants were included in the analysis, consisting of 140 individuals with comorbid depression and stroke and 674 individuals without comorbidity. The latter group included participants with only depression, only stroke, or neither condition (Figure 1).

### 2.2 Nutritional Intake Assessment

The collection of dietary intake data by the NHANES protocol involved two 24‐h dietary recall interviews conducted on non‐consecutive days. The first recall session was held in person at the Mobile Examination Center (MEC), followed by a telephone recall 3–10 days later. The nutrient intake values from both recalls were averaged to better estimate accuracy and lessen day‐to‐day differences. This approach is in line with the U.S. usual‐intake methodology, utilizing repeated recalls to diminish within‐person variability at the population level. Using the USDA Automated Multiple‐Pass Method (AMPM), a validated five‐step technique, trained interviewers conducted all dietary interviews to boost recall accuracy and reduce reporting bias (Blanton et al. 2006; Ahluwalia et al. 2016; Rhodes et al. 2013). Despite the fact that two recalls cannot completely substitute for long‐term repeated assessments, this standardized and rigorously executed approach offers robust, nationally representative estimates that are widely accepted and recognized in nutritional epidemiology (Ahluwalia et al. 2016; Herrick et al. 2018; Freedman et al. 2014). A total of 46 dietary components were analyzed, encompassing macronutrients (e.g., energy, protein, carbohydrates, fats), vitamins (e.g., thiamin, riboflavin, folate, vitamins B6, B12, C, D, E, and K), minerals (e.g., calcium, iron, zinc, magnesium, copper, sodium, potassium, selenium), and bioactive compounds (e.g., caffeine, theobromine, lycopene, lutein, retinol). Nutrient estimates were derived from the USDA Food and Nutrient Database for Dietary Studies (FNDDS) corresponding to each NHANES cycle. The selection of these variables was based on their recognized or potential significance to neurological and vascular health.

### 2.3 Assessment of the Depression and Stroke

Depression evaluation was conducted with the nine‐item Patient Health Questionnaire (PHQ‐9), a validated tool featured in NHANES. Participants were asked how often they had experienced each of the nine depressive symptoms over the past 2 weeks, with responses ranging from “not at all” (score = 0) to “nearly every day” (score = 3). Scores can vary from 0 to 27, with higher numbers showing more severe symptoms. Consistent with previous findings, this study used a PHQ‐9 score of 10 or more to define depression (Ba et al. 2021; Zhang et al. 2024).

Stroke status was assessed through self‐reported information from the NHANES medical conditions questionnaire. Participants were inquired, “Has a doctor or any health professional ever informed you that you experienced a stroke?” A “yes” response classified participants as having a stroke history. Individuals with missing data on this item or who responded “no” were excluded from the analysis.

In this study, comorbidity was operationally defined as the coexistence of both stroke and depression diagnoses at the time of NHANES survey participation. Self‐reported physician diagnosis was used to determine stroke status, and depression was assessed via the PHQ‐9, with a score of 10 or more marking clinically significant depressive symptoms. Consistent with earlier NHANES studies, this definition identifies the co‐occurrence of both conditions during the same survey cycle, without clarifying if they took place at the same time or one after the other. Importantly, due to NHANES's cross‐sectional nature and the absence of time‐specific diagnostic information, determining the temporal sequence is not possible. From a clinical viewpoint, stroke is typically characterized as a sudden vascular event, whereas depression, particularly when identified through the PHQ‐9, often reflects a chronic or subacute mood condition. Given the distinct courses, it is improbable that both diagnoses arose at the same time. Rather, the two conditions may develop in either direction: depression may precede stroke and act as a risk factor, or stroke may lead to post‐stroke depression. Our definition highlights diagnostic coexistence rather than concurrent onset.

### 2.4 Potential Covariates

In order to maintain uniformity in variable definitions, the extraction of covariates followed NHANES procedures for depression and stroke assessment. The NHANES questionnaire, examination, and laboratory components were the direct sources of all covariates. Age, sex, and race/ethnicity demographic characteristics were extracted from the Demographics Module. Socioeconomic indicators, including education level and the family income‐to‐poverty ratio, were derived from the Demographic and Income Questionnaire. Behavioral factors such as smoking and alcohol consumption were obtained from the Lifestyle Questionnaire. The standard NHANES Body Measures Examination (BMX) protocol provided the height and weight measurements used to calculate BMI. Participants' weight was determined to the nearest 0.1 kg using a calibrated digital scale, and their standing height was measured to the nearest 0.1 cm with a stadiometer, with them barefoot and aligned to the Frankfort horizontal plane. The formula for BMI is weight in kilograms divided by the square of height in meters. Hypertension was defined according to the 2017 ACC/AHA guideline as having an average systolic blood pressure ≥ 130 mmHg or an average diastolic blood pressure ≥ 80 mmHg, based on up to three standardized measurements obtained during the NHANES blood pressure examination (Whelton et al. 2018). Using a calibrated sphygmomanometer, trained health technicians measured blood pressure after participants had rested quietly in a seated position for no less than 5 min. Participants were also considered hypertensive if they reported a physician's diagnosis of hypertension or were currently on antihypertensive medication, besides having measured values. Glucose metabolism status was determined using fasting plasma glucose (FPG) and HbA1c values obtained from the NHANES Laboratory Module, following diagnostic thresholds recommended by the American Diabetes Association (ADA), which NHANES uses for classifying glycemic status. Diabetes was determined by a fasting plasma glucose (FPG) of 126 mg/dL or above, an HbA1c of 6.5% or more, a diagnosis reported by the patient from a physician, or the use of medication for diabetes, whereas prediabetes was identified by an FPG ranging from 100 to 125 mg/dL or an HbA1c level between 5.7% and 6.4% (American Diabetes Association Professional Practice Committee 2022).

### 2.5 Statistical Analysis

The baseline features of participants were described by comorbidity status using procedures that account for survey weights. Continuous variables were displayed using means and their corresponding standard deviations. Prior to making statistical comparisons, we used the Shapiro–Wilk test to assess the distribution of all continuous covariates, discovering that several nutrient variables were right‐skewed. To confirm the strength of group comparisons, both the Student t‐test and the Wilcoxon rank‐sum test were conducted, and they delivered consistent results. Categorical variables were described using weighted frequencies and percentages, with comparisons conducted via the Chi‐square test. To tackle multicollinearity, we calculated the variance inflation factor (VIF) for each independent variable and omitted those with VIF values above 3 after adjusting the degrees of freedom. To identify predictors associated with comorbid depression and stroke, we implemented a structured machine learning pipeline. Feature importance ranking and variable selection were performed using the Boruta algorithm, a wrapper‐based method that builds upon random forests to identify all relevant features by comparing the importance of actual variables with randomly permuted shadow features. Only those features classified as important were retained for subsequent model construction. Given the relatively low prevalence of comorbid cases in the dataset, the Synthetic Minority Oversampling Technique (SMOTE) was applied to the training set to address class imbalance. This approach synthetically generates new minority class instances by interpolating between existing samples, thereby improving model sensitivity to the underrepresented class. Weighted Quantile Sum (WQS) regression was employed to assess the combined impact of nutrient mixtures on the comorbidity of depression and stroke. This method is appropriate for analyzing highly correlated dietary exposures and facilitates the identification of nutrients that contribute most to a mixture effect. Prior to model estimation, all nutrient variables were converted into decile‐based quantiles to ensure they were comparable across scales. To bolster model stability and mitigate overfitting, WQS indices were assessed through repeated bootstrap sampling, involving 100 iterations with a 40/60 training‐validation split for each iteration. Within each bootstrap sample, the algorithm assigned non‐negative weights that added up to one, illustrating the relative role of each nutrient in the mixture index. Due to the potential for nutrients to impact risk in opposite directions, two indices were developed: a WQS‐negative index for nutrients that increase risk and a WQS‐positive index for those with protective potential. In the WQS estimation framework, it is expected that the sum of the reported weights does not necessarily equal one, as these values are averaged rather than derived from a single bootstrap run. To explore how demographic, socioeconomic, and clinical factors could modify the connection between dietary factors and comorbidity of depression, we applied stratified modeling. Specifically, Model A was an unadjusted baseline model serving as a reference point. Model B was a fully adjusted model that incorporated a comprehensive set of covariates, including age, sex, race/ethnicity, educational attainment, family income to poverty ratio, body mass index (BMI), smoking status, drinking status, hypertension, and diabetes. We applied six supervised machine learning algorithms to predict the binary outcome of stroke‐depression comorbidity. The algorithms comprised ensemble methods (Random Forest, LightGBM, XGBoost), an instance‐based learner (K‐Nearest Neighbors), a probabilistic classifier (Naive Bayes), and a kernel‐based discriminative approach (Support Vector Machine). The predictive performance of all models was evaluated systematically using established classification metrics, including the area under the curve (AUC), sensitivity, specificity, accuracy, precision‐recall area under the curve (PR‐AUC) and Fβ‐score. The model with the best AUC performance was selected for interpretation. To boost interpretability and transparency, the optimal model was analyzed using SHAP and LIME. By using these strategies, we were able to recognize important dietary factors impacting comorbidity risk and see how feature contributions changed across different subpopulations.

## 3 Results

### 3.1 Baseline Characteristics

Table S1 contains detailed comparisons of baseline characteristics. In this analysis, 814 participants were included, and 140 were diagnosed with comorbid depression and stroke. Individuals with comorbidities were notably younger (64.5 ± 8.37 years) and had a lower family income‐to‐poverty ratio (1.69 ± 1.18) compared to those without comorbidities (69.5 ± 8.91 years and 2.16 ± 1.41, respectively). Notable differences in nutritional intake were also observed; participants with comorbid conditions had significantly lower intakes of thiamin (1.28 ± 0.71 vs. 1.41 ± 0.76 mg), vitamin B6 (1.57 ± 1.58 vs. 1.75 ± 1.15 mg), total folate (303.58 ± 217.11 vs. 336.69 ± 208.50 μg), added vitamin B12 (0.51 ± 2.40 vs. 0.76 ± 1.88 μg), and vitamin C (61.28 ± 86.10 vs. 80.09 ± 89.25 mg). There were also statistically significant differences in sex distribution, with a higher proportion of females among those with comorbidities (55.00% vs. 45.85%, *p* = 0.048), and smoking status, with a greater prevalence of current smokers in the comorbid group (37.14% vs. 19.14%, *p* < 0.001). No major differences were observed in BMI, drinking status, hypertension, or glucose metabolism status.

### 3.2 Joint Nutrient Effects on the Risk of Comorbid Depression and Stroke via WQS Regression

Using WQS regression, we evaluated the overall effects of dietary nutrients on the risk of comorbid depression and stroke, by constructing separate indices for nutrient mixtures with negative and positive associations. By accounting for collinearity among exposures, this method identifies the relative role of each component in the mixtures. Odds ratios (OR) with 95% confidence intervals (CI) are used to report the results. Adjustments were made in the models for relevant sociodemographic and health‐related factors. Table S2 shows that neither the WQS‐negative nor the WQS‐positive index had a significant association with the risk of comorbidity. Both the WQS‐negative index and the WQS‐positive index exhibited non‐significant inverse relationships, with OR of 0.84 (95% CI: 0.62–1.14, *p* = 0.252) and 0.88 (95% CI: 0.71–1.09, *p* = 0.236), respectively. In the WQS‐negative model (Table S3), alcohol (weight = 0.392), alpha‐carotene (weight = 0.191), and added vitamin B12 (weight = 0.097) emerged as the predominant contributors. In contrast, the WQS‐positive model (Table S4) identified theobromine (weight = 0.206), vitamin E (weight = 0.194), and caffeine (weight = 0.122) as the primary components. Although the overall connections were not statistically significant, specific weight patterns related to nutrients were identified.

### 3.3 Development and Validation of a Model to Predict Comorbid Diseases

In response to the class imbalance between participants with and without comorbid depression and stroke, the training data underwent processing with the SMOTE. Figure S1 demonstrates that this process successfully balanced the class distribution by creating synthetic minority class instances, thereby enhancing the model's capability to learn patterns for comorbidity detection. The Boruta algorithm was used on the complete set of candidate features to pinpoint the most informative variables linked to the risk of comorbid depression and stroke. Figure S2 uses orange to highlight important features and blue for shadow features. Of the 32 potential predictors, 31 were confirmed as crucial features, while alpha‐tocopherol was not included due to its Z‐score not exceeding the shadow attributes. The confirmed features included a broad range of demographic, socioeconomic, clinical, and nutritional variables: age, smoking status, sex, education level, diabetes, race/ethnicity, family income‐to‐poverty ratio, caffeine intake, hypertension, vitamin B1, iron, vitamin B12, vitamin B2, lycopene, drinking status, sodium, vitamin C, theobromine, moisture, BMI, added vitamin B12, zinc, vitamin D, calcium, copper, and vitamin E.

**FIGURE 2:**
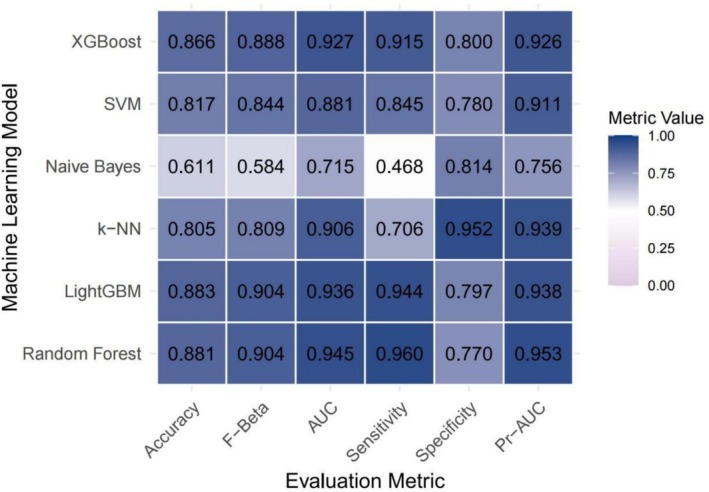
Heatmap of performance metrics across machine learning models (covariate‐adjusted model). AUC, area under the curve; k‐NN, k‐Nearest Neighbors; LightGBM, Light Gradient Boosting Machine; Pr‐AUC, precision‐recall area under the curve; SVM, Support Vector Machine; XGBoost, eXtreme Gradient Boosting.

**FIGURE 3:**
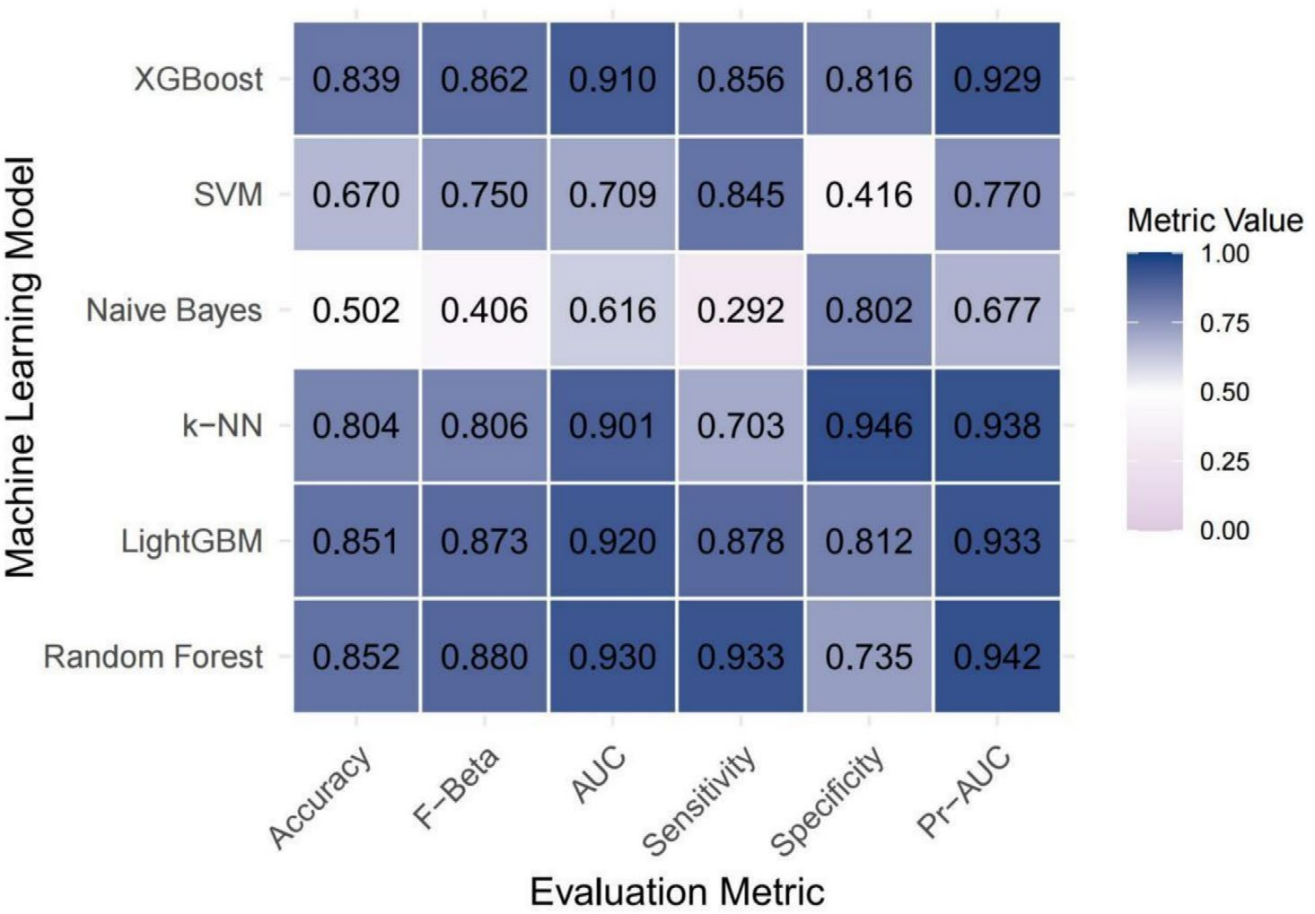
Heatmap of performance metrics across machine learning models (unadjusted model). AUC, area under the curve; k‐NN, k‐Nearest Neighbors; LightGBM, Light Gradient Boosting Machine; Pr‐AUC, precision‐recall area under the curve; SVM, Support Vector Machine; XGBoost, eXtreme Gradient Boosting.

**FIGURE 4:**
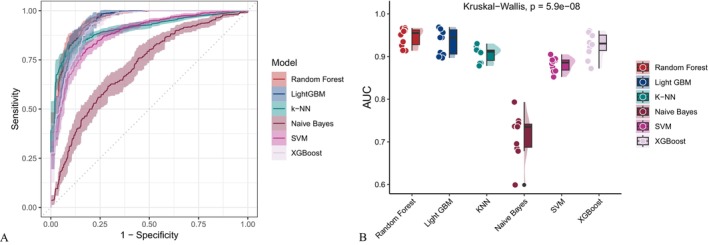
ROC curve comparison across classifiers using covariate‐adjusted model data. AUC, area under the curve; LightGBM, Light Gradient Boosting Machine; ROC, receiver operating characteristic.

**FIGURE 5:**
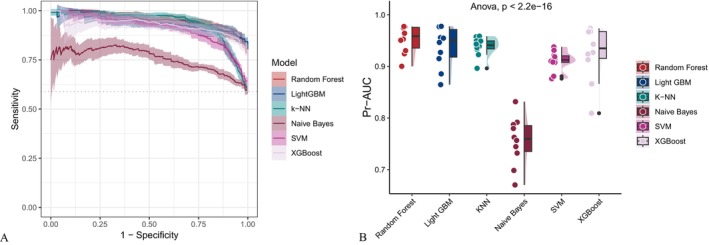
PR curve analysis for covariate‐adjusted model data. Statistically significant differences were observed across models (ANOVA *p* < 2.2^−16^). ANOVA, analysis of variance; PR, precision‐recall.

**FIGURE 6:**
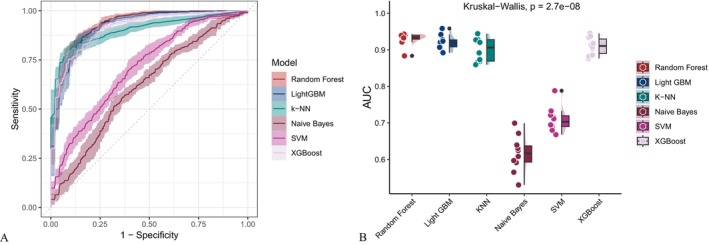
ROC curves generated using the unadjusted dataset. Random Forest and LightGBM achieved the highest AUC values. Kruskal–Wallis test *p* = 2.7^−8^. AUC, Area Under the Curve; LightGBM, Light Gradient Boosting Machine; ROC, receiver operating characteristic.

**FIGURE 7:**
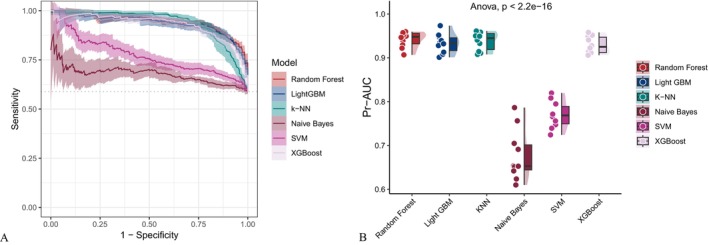
PR curve analysis for the unadjusted dataset. Statistically significant differences were observed across models (ANOVA *p* < 2.2^−16^). ANOVA, analysis of variance; PR, precision‐recall.

As illustrated in Figure 2, covariate‐adjusted models exhibited enhanced performance across multiple evaluation metrics, including the AUC, sensitivity, specificity, accuracy, Fβ score and precision‐recall area under the curve (PR‐AUC) compared to the unadjusted baseline models (Figure 3). While support vector machines (SVM) and gradient boosting methods such as XGBoost and LightGBM exhibited consistently strong performance, the Random Forest model yielded the highest AUC among all evaluated algorithms, irrespective of whether demographic, socioeconomic, and clinical covariates were adjusted. The AUC values reached 0.945 and 0.930 for the adjusted and unadjusted models, respectively. The receiver operating characteristic curve (ROC) (Figure 4) and precision‐recall curve (Figure 5) further confirmed these findings. Statistical comparisons revealed significant differences in model performance before and after oversampling (Kruskal–Wallis test for AUC: *p* = 5.9 × 10^−8^; ANOVA for PR‐AUC: *p* < 2.2 × 10^−16^). In contrast, models lacking covariate‐adjusted (Figures 6 and 7) showed diminished sensitivity and precision, especially concerning minority cases, which highlights the necessity of class‐balancing strategies in this context.

In Figure S3, the overall classification accuracy of six machine learning models is presented under two analytical conditions: Figure S3A reflects models adjusted for demographic, socioeconomic, and clinical covariates, while Figure S3B shows results without such adjustments. Random Forest and LightGBM consistently delivered the best classification accuracy across both scenarios, whereas Naïve Bayes and SVM had lower accuracy, particularly without adjustments. As illustrated in Figure S4, the Fβ score, which represents the harmonic mean of precision and recall, further underscored the superior performance of ensemble models. After adjusting for demographic, socioeconomic, and clinical covariates (Figure S4A), Random Forest achieved the highest Fβ score, and even without covariate adjustment (Figure S4B), it continued to perform best. Figure S5 demonstrates that Random Forest maintained the highest sensitivity in both adjusted and unadjusted models, as revealed by the sensitivity analysis. With demographic, socioeconomic, and clinical variables controlled (Figure S5A), Random Forest exhibited nearly flawless sensitivity, and the model continued to excel even without covariate adjustments (Figure S5B). As shown in Figure S6, model‐specific specificity values varied across adjustment settings. When demographic, socioeconomic, and clinical covariates were included in the model (Figure S6A), KNN demonstrated the highest specificity among all models, followed by Random Forest and LightGBM, both of which also exhibited consistently high and stable performance. In contrast, eliminating covariate adjustment (Figure S6B) led to a notable decline in SVM's specificity, while KNN continued to surpass other models in minimizing false positives.

**FIGURE 8:**
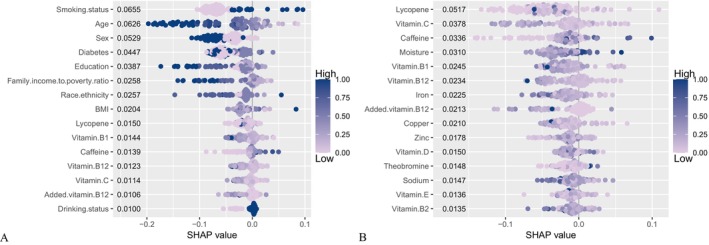
SHAP beeswarm plot showing the impact of each feature on the model output across all individuals. Each dot represents an individual observation for a given nutrient. Dot color reflects the original feature value, with red indicating higher nutrient levels and blue indicating lower levels. The position along the x‐axis corresponds to the SHAP value, which quantifies the direction and magnitude of the feature's contribution to the model prediction. Nutrients for which red dots cluster on the positive side of the x‐axis indicate that higher values increase the predicted comorbidity risk, whereas those showing red dots on the negative side indicate a protective association. The horizontal spread of dots demonstrates inter‐individual variability in the strength of each nutrient's influence. Nutrients are ordered by their mean absolute SHAP values, with those contributing most to the model displayed at the top. SHAP, SHapley Additive exPlanations.

Figure 8 illustrate the SHAP‐based feature importance analyses for two different model setups: Figure 8A includes demographic, socioeconomic, and clinical adjustments, while Figure 8B excludes these adjustments. In Figure 8A, a SHAP beeswarm plot is displayed, demonstrating the relative importance and directional influence of each predictor on the model's results. In the SHAP beeswarm plot, each point signifies a single observation for a specific nutrient. The color gradient reflects the original feature values, where red denotes higher nutrient levels and blue denotes lower levels. The SHAP value, shown horizontally, reflects both the direction and magnitude of how each nutrient contributes to the predicted comorbidity risk. Red dots appearing on the positive side of the x‐axis signify that elevated nutrient levels increase the predicted comorbidity risk, whereas blue dots on the negative side suggest that reduced levels lower the risk. Conversely, when red dots appear predominantly on the negative side, higher nutrient levels are associated with a lower predicted risk. The horizontal spread of dots reflects inter‐individual heterogeneity in the strength of each nutrient's influence. Nutrients are ordered by their mean absolute SHAP values, with those contributing most to the model displayed at the top. Higher intake of added vitamin B12, vitamin C, vitamin B12, vitamin B1, and lycopene was primarily associated with negative SHAP values. In contrast, elevated caffeine intake, frequent alcohol use, BMI, older age, male sex, diabetes, and current smoking corresponded to higher SHAP values. In addition, a reduced family income‐to‐poverty ratio and particular race/ethnicity categories, which are generally indicative of lower socioeconomic status, demonstrated positive SHAP values when in the low range (blue). In the SHAP beeswarm plot presented in Figure 8B, where the model lacks adjustments for demographic, socioeconomic, and clinical covariates, nutritional factors continue to be significant contributors to the model's predictions. Higher intake of vitamin B2, vitamin E, sodium, theobromine, vitamin D, zinc, copper, added vitamin B12, iron, vitamin B12, vitamin B1, vitamin C, and lycopene was primarily associated with negative SHAP values. Conversely, elevated levels of moisture and caffeine corresponded to positive SHAP values. In Figure S7A, which displays mean absolute SHAP values, smoking status, age, and sex emerged as the most influential predictors in the covariate‐adjusted model. In contrast, dietary variables such as drinking status, added vitamin B12, and vitamin C were associated with relatively lower SHAP values. In Figure S7B, where demographic and clinical covariates were excluded from the model, nutritional variables dominated the rankings. In the absence of adjustments, the model predictions were most strongly influenced by lycopene, vitamin C, caffeine, and moisture, which had the highest SHAP values.

Figure S8 (adjusted for covariates) and Figure S10 (without adjustments) show model predictions for the same person under two different classification results. On the left side of each figure, there is a prediction of “no comorbidities,” and on the right side, a prediction of “comorbidities” is shown. In both models, a symmetrical pattern was evident: when an individual was predicted to lack comorbidities, the feature contributions were positive, resulting in a higher predicted probability (*f*(*x*) = 0.867 in Figure S8 and *f*(*x*) = 0.839 in Figure S10). In contrast, when comorbidities were anticipated, the same features negatively influenced the prediction score, resulting in *f*(*x*) values of 0.133 and 0.161. In Figures S9 and S11, SHAP dependence plots are shown for models that are adjusted for covariates and those that are not, respectively. Each plot depicts how a single predictor marginally affects the model output across its range of values. In the adjusted model (Figure S9), features such as lycopene, vitamin B1, vitamin B12, caffeine, and added vitamin B12 exhibited relatively linear and symmetric relationships with SHAP values, suggesting stable contributions to the predicted probability of comorbidities. In contrast, the unadjusted model (Figure S11) demonstrated more variable and in some cases nonlinear patterns, particularly for lycopene, vitamin C, and moisture.

Figure S12 through Figure S13 display LIME‐based local explanations for a representative individual (Case 87) under the covariate‐adjusted model. The model assigned a 76% probability of being classified as without comorbidities and a 24% probability of being classified as with comorbidities (Figure S12). For the “no comorbidities” prediction, features such as higher vitamin B1, zinc, and vitamin C levels contributed positively, while lower added vitamin B12, lower education, and reduced alpha‐tocopherol exerted a negative influence (Figures S12 and S13). Conversely, when the model considered the alternative classification (“comorbidities”), the same features exerted opposite directional effects. Figure S14 through Figure S15 present the LIME‐based local explanations for the same individual (Case 87) under the unadjusted model. The model predicted a 79% probability of being classified as without comorbidities and a 21% probability of being classified as with comorbidities (Figure S14). Key features contributing to the “no comorbidities” prediction included elevated levels of vitamin C, vitamin B1, zinc, and sodium, while low values of added vitamin B12 and vitamin B12 also supported this classification. When the same individual was hypothetically assigned the opposite label (“comorbidities”), these features exerted reverse directional effects, mirroring their roles across the classification boundary (Figures S14 and S15). Compared with the covariate‐adjusted LIME results (Figures S14 and S15), the unadjusted model showed similar contributing features but with slightly altered thresholds and weights.

## 4 Discussion

Our findings demonstrate the value of using interpretable machine learning approaches to explore the complicated relationships between dietary nutrient intake and the comorbidity of depression and stroke within a nationally representative population. Rather than identifying statistically significant associations at the mixture level, our analyses highlighted a subset of individual nutrients, including alcohol, alpha‐carotene, added vitamin B12, theobromine, and vitamin E, which contributed disproportionately within nutrient indices. Importantly, when model interpretability techniques were applied, vitamin B1, vitamin B12, vitamin C, zinc, and caffeine consistently emerged as key predictors, suggesting that these micronutrients may be critical markers of neurovascular vulnerability. Incorporating SHAP and LIME allowed us to go beyond predictive accuracy and understand how different nutrient intake patterns could influence comorbidity risk. The Random Forest algorithm, which achieved the highest discriminative performance (AUC = 0.945), offered a robust framework for prediction; yet its clinical value lies less in the raw performance metrics and more in the transparency afforded by interpretable AI. The insights suggest that models that are explainable can help prioritize dietary factors for future exploration, thus narrowing the gap between statistical predictions and clinical applicability. From a translational angle, our results point to the potential of using nutritional indicators alongside standard risk stratification methods for individuals experiencing both depression and stroke. Even though the current data are cross‐sectional and do not establish causality or temporal sequence, the consistency of nutrient signals across various analytical frameworks strengthens the justification for prospective validation. In particular, future longitudinal cohorts and dietary intervention trials are needed to determine clinically meaningful thresholds, quantify effect sizes, and evaluate whether modification of specific nutrient intakes can reduce the burden of this comorbidity.

Our findings can be better understood by examining them alongside existing work on nutrition‐related health prediction models. The use of machine learning in dietary data is still emerging, yet an increasing number of studies are applying AI/ML methods to predict health outcomes from nutritional and lifestyle inputs. In their review, Ferrario et al. discuss the potential and hurdles of applying machine learning to combine dietary, omics, and lifestyle elements in personalized nutrition models (Ferrario and Gedrich 2024). The necessity of variable selection and regularization is stressed due to the “*p* > *n*” challenge, where predictors outnumber samples, aligning with our decision to implement Boruta and feature selection prior to model fitting. According to Kirk et al., machine learning approaches can support nutrition researchers in dealing with complex dietary data, offering methodological advice on preprocessing variables, cross‐validation, and interpretation (Kirk et al. 2022). In more practical prediction studies, multi‐dimensional health and dietary indicators are used to predict cognitive outcomes, illustrating how nutrition data can enhance models predicting neurological health. The study, although not specifically focused on comorbid depression and stroke, shows the potential of combining dietary and health biomarkers to forecast neuropsychiatric results (Verma et al. 2025). The review by Agrawal et al. explores AI‐driven innovations in personalized nutrition, highlighting how interpretable models might be more effectively applied to real‐world dietary advice (Agrawal et al. 2025). They mention that one of the main challenges is the transparency of algorithms, as models that remain opaque often impede their clinical implementation. Compared to these prior works, our study contributes in several ways. Firstly, by concentrating on the co‐occurrence of depression and stroke, we examine a less studied area at the intersection of neurology, psychiatry, and nutrition. Secondly, we integrate explainable methods like SHAP and LIME with strong machine learning frameworks to achieve both predictive accuracy and interpretability, thus connecting predictive performance with actionable insights. Thirdly, while many published ML‐nutrition studies are constrained to macronutrients or small biomarker panels, we analyze a relatively broad panel of 46 dietary components, which offers a more comprehensive view of nutrient patterns associated with mental and vascular health.

While acknowledging this, it's important to understand that not every analytic approach led to the same conclusions. There was a clear inconsistency between the results of WQS regression and those of the machine learning models. While WQS regression did not find a statistically significant effect from the mixture, machine learning techniques exhibited excellent predictive performance. The overall mixture effect was not statistically significant according to WQS regression, yet machine learning approaches displayed strong predictive results. This difference reflects the distinct goals and assumptions of the two methods. WQS regression is primarily designed to test for a constrained, weighted linear effect of the entire nutrient mixture, which may yield null results when associations are nonlinear, heterogeneous, or concentrated within a subset of nutrients. Recent methodological work has highlighted these limitations and proposed extensions such as penalized weights to better capture complex or bidirectional mixture effects. In comparison, machine learning algorithms are fine‐tuned for prediction and can flexibly manage nonlinear relationships, interactions, and complex dependencies among dietary variables. The accuracy achieved by our machine learning models reveals that there are important signals within the nutrient data, even if they do not appear as a consistent mixture effect under the WQS framework. Similar applications of explainable AI in public health and clinical prediction, including stroke risk modeling and obesity determinants, have further underscored the value of SHAP and LIME in generating interpretable insights that complement traditional regression approaches. Taken together, these findings highlight the complementary roles of traditional mixture regression and explainable machine learning: the former provides interpretable statistical inference under predefined assumptions, whereas the latter offers robust prediction and hypothesis generation in the context of complex dietary exposures.

In machine learning models, SHAP values help identify the impact of each feature on the predictions. Understanding these core theoretical ideas is important in the scenario of depression and stroke co‐occurrence. For instance, in a study investigating the prediction of comorbid cardiovascular disease and cancer based on dietary antioxidant intake (Qi et al. 2025), SHAP analysis derived from the LightGBM model revealed that naringenin, magnesium, theaflavin, kaempferol, hesperetin, selenium, malvidin, and vitamin C were the most influential contributors. This exemplifies how SHAP can uncover key components in complex models for predicting comorbidities. SHAP was also used in another study that explored stroke risk factors and dietary protective elements based on the NHANES database (Wu, Yu, et al. 2025). With an AUC of 82%, a prediction model was developed, and SHAP values analyzed the influence of factors such as age, income level, and cholesterol, which resulted in a scoring scale with an AUC of 79%. SHAP's application in studies of related comorbidities shows its potential to identify the importance of various factors in predicting the comorbidity of depression and stroke. LIME is an effective tool for understanding prediction models. It assists in revealing the factors that contribute to the model's predictions during the evaluation of depression in stroke patients. In a study that developed and validated a clear predictive model for depression among stroke patients (Zuo and Yang 2025), the SHAP method was applied to assess feature importance after the model was constructed with machine learning algorithms. In a similar fashion, LIME can be employed to increase the local interpretability of model predictions. For instance, in research focused on creating an explainable AI model for predicting strokes using EEG signals (Islam et al. 2022), XAI methods like LIME were employed to elucidate the model's decision‐making process. LIME identified the spectral delta and theta features as local influences on stroke prediction. This indicates that in the field of depression and stroke prediction, LIME can play a role in highlighting the significant features that contribute to the model's output, enabling a better understanding of how the model arrives at its predictions and potentially guiding more targeted interventions.

When it comes to interpreting medical data, SHAP and LIME each have their own unique characteristics. The LIME algorithm demonstrated superior performance in classification accuracy compared to other feature selection methods in most fNIRS dataset cases, according to a comparative study (Shin 2023). However, SHAP and LIME, which are used for individual feature analysis, face limitations due to their reliance on perturbations. They analyze how AI models handle unexpected changes in particular feature values, which may not accurately depict real‐world scenarios (Contreras et al. 2024). To tackle this issue, an adapted version of LIME and SHAP was developed to perform group perturbations, enhancing both explainability and realism and minimizing noise in interpretability plots. Understanding these differences in the context of depression and stroke comorbidity can guide researchers in picking the appropriate technique based on the data's nature and the questions being investigated. For example, when a detailed and immediate understanding of how features contribute to a prediction is necessary, LIME might be preferred, while SHAP could be more appropriate for assessing feature importance on a global level in the model.

The relationship between depression and stroke has become a more prominent research topic over the years. A study investigating trends in outpatient depression treatment among stroke survivors in the United States between 2004 and 2017 reported that the proportion receiving such treatment was 17.7% in 2004–2005 and declined slightly to 16.0% in 2016–2017 (Dong et al. 2022). An analysis of 18‐year trends in stroke mortality and the prognostic role of comorbidities identified substantial improvements in both short‐ and long‐term mortality for all stroke types between 1994 and 2011. The burden of comorbidity notably emerged as a strong indicator of mortality across both time horizons (Schmidt et al. 2014). Between 1990 and 2021, there has been an upward trend in the global disease burden of depressive disorders among young people, with females usually having higher prevalence rates and DALYs (Yang et al. 2024). These trends from history underscore the transformation in depression and stroke comorbidity, encompassing treatment patterns and the disease burden as they have changed over time. The latest epidemiological data give an insight into the frequency and consequences of having both depression and stroke. The study, which included several cohorts, showed a significant association between stroke and an increased likelihood of depressive symptoms. According to the meta‐analysis, the pooled hazard ratio was 1.35 (95% CI: 1.26–1.44), which implies that stroke survivors have an increased risk of developing depressive symptoms (Le et al. 2023). Depression was found to be associated with more than a twofold increase in stroke odds (OR = 2.41; 95% CI: 1.78–3.27) in a study of middle‐aged women, though this association lessened after adjusting for various factors (OR = 1.94; 95% CI: 1.37–2.74) (Jackson and Mishra 2013). Post‐stroke depression (PSD) was observed in 29.0% (95% CI, 25.2%–32.8%) of patients 1 year after experiencing a minor stroke (Shi et al. 2015). The study highlights the heavy toll depression takes on this population and underscores the mutual relationship between depression and stroke risk. These findings underscore the substantial burden of depression in this population and highlight the bidirectional relationship between depression and stroke risk.

Depression and stroke are interconnected by multiple biological pathways. A study investigating the bidirectional relationship and shared genetic architecture between depression and stroke using NHANES data and bioinformatic analysis identified 41 commonly upregulated genes and eight commonly downregulated genes associated with both ischemic stroke and major depressive disorder (Yang et al. 2023). The enrichment analysis indicated that these common genes were primarily associated with immune response and pathways related to immunity. A different study investigating various biomarkers for forecasting post‐stroke depression identified increased levels of growth differentiation factor‐15, anticardiolipin antibodies, antiphosphatidylserine antibodies, and matrix metalloproteinase‐9 as each independently associated with an increased risk of depression following ischemic stroke (Che et al. 2021). The study of various biomarkers revealed a clear dose–response relationship, with the likelihood of post‐stroke depression rising as the number of elevated biomarkers increased. With multivariable adjustments, the odds of developing depression were 6.52 times higher (95% CI, 2.24–18.95) in patients showing increases in all four biomarkers, compared to those without such increases. The findings imply that biological pathways involving the immune system and biomarkers are important in the comorbidity of depression and stroke. Neuroinflammation is a developing research area concerning the coexistence of depression and stroke. Innate immunity‐induced neuroinflammation might encourage the onset and development of PSD (Xiao et al. 2024). Peripheral inflammation post‐stroke could start a damaging cycle of neuroinflammation by activating innate immune pathways in the central nervous system, possibly aiding in the development of PSD. Studies have investigated the variations in macrophages and the neuroinflammation triggered by cytokines in the context of major depressive disorder (Dey and Hankey Giblin 2018). Interleukin‐6 (IL‐6), tumor necrosis factor‐alpha (TNFα), and interleukin‐1 beta (IL‐1β) are three significant cytokines that have been widely investigated concerning the causes of major depressive disorder. A bibliometric analysis conducted between 2004 and 2023 on neuroinflammation in depression highlighted the NLRP3 inflammasome, microglia, tumor necrosis factor‐alpha (TNF‐α), and brain‐derived neurotrophic factor (BDNF) as central components in the underlying pathogenic mechanisms (Shi et al. 2025). Targeting neuroinflammation may provide a therapeutic avenue for the combined condition of depression and stroke, according to these findings.

In research involving stroke patients, five machine learning algorithms were used to create a model predicting depression (Zuo and Yang 2025). With an AUC of 0.746 on the ROC curve and an accuracy of 0.834, the XGBoost model exhibited the highest discriminative capability in the test set. In another study, the goal was to design machine learning models to predict the emergence of post‐stroke depression (PSD) based on real‐world data (Chen et al. 2023). The average specificity and sensitivity of the four models were 0.83–0.91 and 0.30–0.48, respectively. In the diagnosis of depression, machine learning can also be integrated with EEG‐based biomarkers. For example, a review examined the integration of machine learning and deep learning models into EEG‐based depression diagnosis, highlighting their potential to enhance diagnostic accuracy and support the development of personalized therapeutic strategies through advanced EEG data analysis (Boby and Veerasingam 2025). Machine learning's potential to refine the diagnostic accuracy of depression and stroke comorbidity is illustrated by these applications. The diagnostic accuracy of depression and stroke comorbidity can be significantly enhanced by SHAP and LIME. In a study on predicting residual axillary lymph node metastases in triple‐negative breast cancer, the SHAP method was used to construct a radiomics nomogram, which achieved good predictive efficacy with an AUC of 0.922 (95% CI, 0.890–0.954) in the training cohort and 0.904 (95% CI, 0.853–0.955) in the validation cohort (Yao et al. 2025). SHAP gave more detailed insights into the role of each feature in the model's outcomes. In diagnosing depression and stroke, SHAP could be employed to detect crucial features, much like its use in the breast cancer study. LIME, on the other hand, can provide local interpretability. For instance, in research on an explainable AI model for predicting strokes using EEG signals, LIME was employed to clarify the model's behavior and provide local interpretation around the prediction (Islam et al. 2022). By understanding the role of individual features in diagnostic decisions, local interpretability can improve the overall accuracy of diagnostics.

Machine learning is driving the development of personalized treatment strategies, which show promise for managing depression and stroke comorbidity. In other disease contexts, such as pancreatic cancer, machine learning‐based approaches have been employed to map multi‐omics alterations in m6A readers, construct prognostic scoring models, and identify potential drug targets for high‐risk patients (Chen et al. 2024). In breast cancer scenarios, machine learning algorithms were devised to anticipate individual treatment impacts and support the creation of customized treatment approaches (Ren et al. 2024). By examining patient‐specific data like genetic, biomarker, and clinical information, machine learning could help develop personalized treatment plans for those suffering from both depression and stroke. This may involve predicting which patients are more likely to respond favorably to certain antidepressants or psychotherapies, or which treatment combinations would be most effective based on unique individual features.

Using SHAP and LIME for medical predictions brings up ethical issues, highlighting the necessity of considering patient perspectives and involvement in healthcare predictive models (Markham 2025). Significant ethical concerns about patient autonomy, safety, and justice arise for mHealth applications that may utilize SHAP or LIME for diagnostic support (Laflamme et al. 2019). When it comes to diagnosing and treating depression and stroke comorbidity, SHAP and LIME should be used ethically to ensure patient data is responsibly managed and that their interpretability does not lead to misinterpretation or discrimination. In the field of artificial intelligence research on hemorrhagic stroke, the overall reporting quality of published AI and machine learning studies remains suboptimal, characterized by limited transparency and poor reproducibility (Lim et al. 2024). Despite the good performance of the interpretable model for mental health treatment prediction, issues such as ensuring explainability, confidence, and robustness of predictions persist (Kelly et al. 2025). In depression and stroke scenarios, the challenges are heightened due to the complex nature of their comorbidity. Models are required to predict this comorbidity accurately and deliver results that are interpretable by clinicians and patients. Improving the reporting of study details, expanding the use of model examination techniques, and creating models that are more naturally interpretable are required. In psychiatry, challenges such as the curse of dimensionality, data quality, the black box problem, hyperparameter tuning, external validation, class imbalance, and data representativeness need to be addressed to improve the reliability of machine learning models (Ostojic et al. 2024).

Our findings may hold potential significance for public health and clinical practice. The consistent identification of specific micronutrients, including vitamin B1, vitamin B12, vitamin C, zinc, and caffeine, suggests that dietary patterns may play a role in shaping vulnerability to the comorbidity of depression and stroke. The cross‐sectional data do not allow for causal inference, but the results provide evidence that can generate hypotheses for future longitudinal and intervention studies. With additional validation, nutrient‐based risk profiles might be utilized in screening tools to identify at‐risk groups, inform personalized dietary counseling, and back precision nutrition strategies. By integrating this evidence into preventive guidelines at the population level, we could potentially reduce the incidence of neurovascular and psychiatric comorbidities, showcasing the broader benefits of combining nutritional epidemiology with explainable artificial intelligence.

This study has several limitations that warrant careful consideration. First, the cross‐sectional format of NHANES prevents it from making definitive claims about whether nutrient intake leads to or follows the occurrence of depression and stroke. Thus, the links observed in this study should be regarded as exploratory and for the purpose of generating hypotheses, not confirming them. In addition, as the analytic sample was specific to the U.S. population, external validation in other demographic and geographic regions is essential. To determine whether the identified biomarkers are causal drivers, intermediates, or correlates of comorbidity, future longitudinal, mechanistic, and mediation studies are necessary. Second, the limited number of comorbid cases reduces statistical power and could impact the robustness and generalizability of the machine learning models. Third, the lack of follow‐up data with a temporal structure made temporal splitting unfeasible, and the small sample size also constrained the practical application of bootstrap resampling. Fourth, the cross‐sectional format of NHANES does not allow for conclusions about the order of occurrence between stroke and depression. Both diagnoses were identified during the survey, yet it remains ambiguous whether depression was an antecedent to the stroke or a subsequent complication. Given that strokes occur abruptly and depression is often a persistent condition, their simultaneous occurrence is improbable. Our definition highlights diagnostic coexistence rather than concurrent onset. Longitudinal studies are needed to shed light on these temporal dynamics and underlying causal mechanisms. Fifth, even though the random forest classifier achieved a high discriminative performance with an AUC of 94.5%, NHANES lacks the necessary longitudinal outcomes, clinical workflows, and decision‐making processes to assess its clinical impact. Sixth, the NHANES dietary exposure data were derived from two separate 24‐h dietary recalls. Although this method is widely used and generally validated in nutritional epidemiology, it remains susceptible to recall bias and day‐to‐day variation, which are inherent to self‐reported dietary assessments. In addition, biomarker validation was not feasible in the present study, as NHANES provides biomarker data for only a subset of nutrients, whereas our analysis considered a broader panel of 46 dietary components.

## 5 Conclusion

The research underscores the role of explainable machine learning in decoding the complex interplay between dietary nutrients and the comorbidity of depression and stroke. Although the associations at the mixture level were not statistically significant, nutrients like vitamin B1, vitamin B12, vitamin C, zinc, and caffeine consistently proved to be influential predictors. The integration of interpretable methods such as SHAP and LIME enabled the analysis to go beyond predictive performance, offering transparent insights into both overarching trends and individual risk profiles. The study emphasizes the potential benefits of merging nutritional data with interpretable AI to form a foundation for individualized risk assessments and to steer future longitudinal and intervention research targeting the reduction of neurovascular disease burden.

## Author Contributions

**Miaomiao Hou:** conceptualization, investigation, funding acquisition, writing – original draft, methodology, validation, visualization, writing – review and editing, software, formal analysis, project administration, data curation, supervision, resources. **Hongwei Liu:** conceptualization, investigation, funding acquisition, writing – original draft, methodology, validation, visualization, writing – review and editing, software, formal analysis, project administration, data curation, supervision, resources. **Minghui Wu:** conceptualization, investigation, methodology, validation, formal analysis, software, data curation, visualization, writing – original draft, writing – review and editing, project administration, supervision, resources. **Peng Wei:** conceptualization, investigation, methodology, validation, formal analysis, software, data curation, visualization, writing – original draft, writing – review and editing, project administration, supervision, resources. **Haixia Fan:** conceptualization, investigation, methodology, validation, formal analysis, software, data curation, visualization, writing – original draft, writing – review and editing, project administration, supervision, resources.

## Funding

The Taiyuan Bureau of Science and Technology, Science, Technology, and Innovation Medical Center (Grant No: 202207) offered extra funding for the study.

## Ethics Statement

This study's parts that involved human participants, human materials, or human data were performed in line with the Declaration of Helsinki and were sanctioned by the NCHS Ethics Review Board. Written consent was secured from the patients/participants for their participation in the study.

## Consent

The authors have nothing to report.

## Conflicts of Interest

The authors declare no conflicts of interest.

## Supporting information


**Table S1:** Baseline characteristics of participants.
**Table S2:** Associations between WQS‐derived nutrient indices and risk of comorbid depression and stroke.
**Table S3:** Nutrient weights from the WQS‐negative model for comorbid depression and stroke.
**Table S4:** Nutrient weights from the WQS‐positive model for comorbid depression and stroke.

**Figure S1:** Distribution of class balance before and after SMOTE.
**Figure S2:** Boruta feature importance plot for comorbidity prediction.
**Figure S3:** Accuracy scores of six machine learning models applied to the dataset. Random Forest and LightGBM demonstrated the highest classification accuracy across all models.
**Figure S4:** Fβ scores (*β* = 1) for each classifier, reflecting the harmonic mean of precision and recall Ensemble models outperformed others, with Random Forest achieving the highest Fβ score.
**Figure S5:** Sensitivity (recall) rates across models, indicating the ability to correctly identify individuals at high comorbidity of depression and stroke risk. LightGBM and Random Forest showed the highest sensitivity.
**Figure S6:** Specificity values of the classifiers, measuring true negative rates. SVM showed the highest specificity, whereas ensemble models maintained balanced sensitivity and specificity.
**Figure S7:** SHAP feature importance plot ranking predictors by their average absolute SHAP values. Features such as age, vitamin B1, and diabetes status contributed most to model predictions.
**Figure S8:** SHAP waterfall plot under covariate‐adjusted model for individual‐level prediction.
**Figure S9:** SHAP dependence plots under covariate‐adjusted model.
**Figure S10:** SHAP waterfall plot under unadjusted model for individual‐level prediction.
**Figure S11:** SHAP dependence plots under unadjusted model.
**Figure S12:** LIME explanation summary for covariate‐adjusted model (representative individual).
**Figure S13:** LIME‐derived feature contributions under covariate‐adjusted model (representative individual).
**Figure S14:** LIME‐derived local explanation summary under unadjusted model (representative individual).
**Figure S15:** LIME‐derived feature contributions under unadjusted model (representative individual).

## Data Availability

The data that support the findings of this study are available from the corresponding author upon reasonable request.
