# Supplementary material for: Using SHAP and LIME to Explain Machine Learning Models Predicting Comorbid Depression and Stroke From Daily Dietary Nutrient Intake in a US Population‐Based Study
Source: Food Sci Nutr. 2025 Dec 30;14(1):e71401. doi: 10.1002/fsn3.71401 (PMC12753580; doi:10.1002/fsn3.71401)
Supplement: Supplementary file 1 — Table S1: Baseline characteristics of participants. Table S2: Associations between WQS‐derived nutrient indices and risk of comorbid depression and stroke. Table S3: Nutrient weights from the WQS‐negative model for comorbid depression and stroke. Table S4: Nutrient weights from the WQS‐positive model for comorbid depression and stroke. [file FSN3-14-e71401-s001.docx]

**Supplementary Table 1** Baseline characteristics of participants

| **Characteristic** | **Overall (N=814)** | **Non-comorbidities of (N=674)** | **Comorbidities of (N=140)** | **P value** |
| --- | --- | --- | --- | --- |
| **Age (year)^a^, Mean±SD** | 68.67±9.01 | 69.53±8.91 | 64.55±8.37 | <0.001 |
| **PIR^a,^ Mean±SD** | 2.08±1.38 | 2.16±1.41 | 1.69±1.18 | <0.001 |
| **BMI^a^, Mean±SD** | 29.75±6.58 | 29.55±6.27 | 30.69±7.85 | 0.206 |
| **Sex^b^, n (%)** |  |  |  | 0.048 |
| Female | 386 (47.42%) | 309 (45.85%) | 77 (55.00%) |  |
| Male | 428 (52.58%) | 365 (54.15%) | 63 (45.00%) |  |
| **Race/ethnicityb, n(%)** |  |  |  | 0.482 |
| Mexican | 69 (8.48%) | 55 (8.16%) | 14 (10.00%) |  |
| Other Hispanic | 52 (6.39%) | 40 (5.93%) | 12 (8.57%) |  |
| Non-Hispanic White | 419 (51.47%) | 349 (51.78%) | 70 (50.00%) |  |
| Non-Hispanic Black | 218 (26.78%) | 186 (27.60%) | 32 (22.86%) |  |
| Other Race | 56 (6.88%) | 44 (6.53%) | 12 (8.57%) |  |
| **Smoking status^b^, n(%)** |  |  |  | <0.001 |
| No | 633 (77.76%) | 545 (80.86%) | 88 (62.86%) |  |
| Yes | 181 (22.24%) | 129 (19.14%) | 52 (37.14%) |  |
| **Drinking status^b^, n(%)** |  |  |  | 0.087 |
| No | 126 (15.48%) | 111 (16.47%) | 15 (10.71%) |  |
| Yes | 688 (84.52%) | 563 (83.53%) | 125 (89.29%) |  |
| **Hypertension^b,^ n (%)** |  |  |  | 0.893 |
| No | 171 (21.01%) | 141 (20.92%) | 30 (21.43%) |  |
| Yes | 643 (78.99%) | 533 (79.08%) | 110 (78.57%) |  |
| **Glucose metabolism status^b^, n (%)** |  |  |  | 0.261 |
| NGR | 393 (48.28%) | 332 (49.26%) | 61 (43.57%) |  |
| Pre-DM | 116 (14.25%) | 98 (14.54%) | 18 (12.86%) |  |
| DM | 305 (37.47%) | 244 (36.20%) | 61 (43.57%) |  |
| **Energy^a^, Mean±SD** | 1,782.05±871.92 | 1,777.24±859.07 | 1,805.19±934.24 | 0.954 |
| **Protein^a,^ Mean±SD** | 67.50±35.45 | 67.64±35.26 | 66.82±36.49 | 0.602 |
| **Carbohydrate^a^, Mean±SD** | 217.22±109.03 | 217.14±108.20 | 217.62±113.36 | 0.820 |
| **Total Sugar^a^, Mean±SD** | 97.92±67.83 | 96.78±64.57 | 103.40±81.77 | 0.937 |
| **Dietary fiber^a^, Mean±SD** | 14.67±9.12 | 14.77±8.93 | 14.22±10.02 | 0.102 |
| **Total Fat^a^, Mean±SD** | 70.41±43.36 | 69.87±41.98 | 73.00±49.58 | 0.992 |
| **Saturated fatty acids^a^, Mean±SD** | 22.92±15.18 | 22.69±14.92 | 24.01±16.42 | 0.609 |
| **Monounsaturated fatty acids^a^, Mean±SD** | 25.14±16.19 | 24.96±15.74 | 26.04±18.24 | 0.982 |
| **Polyunsaturated fatty acids^a^, Mean±SD** | 16.06±11.67 | 15.99±11.14 | 16.39±14.00 | 0.456 |
| **Cholesterol^a^, Mean±SD** | 273.37±222.28 | 271.05±215.21 | 284.54±254.16 | 0.801 |
| **Vitamin E as alpha-tocopherol^a^, Mean±SD** | 7.08±5.17 | 7.08 ± 5.04 | 7.06 ± 5.76 | 0.513 |
| **Alpha-tocopherol^a^, Mean±SD** | 0.48±2.61 | 0.46±2.35 | 0.54±3.61 | 0.251 |
| **Retinol^a^, Mean±SD** | 403.53±606.89 | 417.96±654.75 | 334.04±269.74 | 0.169 |
| **Vitamin A^a^, Mean±SD** | 601.22±715.16 | 614.65±751.49 | 536.61±502.06 | 0.110 |
| **Alpha-carotene^a^, Mean±SD** | 339.30±964.18 | 346.77±972.92 | 303.39±923.42 | 0.082 |
| **Beta-carotene^a^, Mean±SD** | 2,161.96±4,028.20 | 2,143.88±3,983.83 | 2,249.04±4,249.11 | 0.157 |
| **Beta-cryptoxanthin^a^, Mean±SD** | 86.96±263.91 | 92.29±286.52 | 61.31±95.29 | 0.124 |
| **Lycopene^a^, Mean±SD** | 4,181.21±8,795.05 | 4,102.36±8,149.57 | 4,560.81±11,431.95 | 0.239 |
| **Lutein+zeaxanthin^a^, Mean±SD** | 1,477.65±3,578.60 | 1,448.26±3,524.90 | 1,619.14±3,836.78 | 0.230 |
| **Thiamin (Vitamin B1)^a^, Mean±SD** | 1.39±0.75 | 1.41±0.76 | 1.28±0.71 | 0.023 |
| **Riboflavin (Vitamin B2)^a^, Mean±SD** | 1.84±1.11 | 1.87±1.12 | 1.74±1.03 | 0.200 |
| **Niacin^a^, Mean±SD** | 20.74±11.43 | 20.96±11.58 | 19.66±10.61 | 0.297 |
| **Vitamin B6^a^, Mean±SD** | 1.72±1.24 | 1.75±1.15 | 1.57±1.58 | 0.008 |
| **Total folate^a^, Mean±SD** | 331.00±210.24 | 336.69±208.50 | 303.58±217.11 | 0.039 |
| **Folic acid^a^, Mean±SD** | 145.25±151.31 | 149.04±148.37 | 126.97±164.05 | 0.197 |
| **Food folate^a^, Mean±SD** | 185.98±120.08 | 187.86±117.88 | 176.89±130.23 | 0.070 |
| **Folate(DFE) ^a^, Mean±SD** | 432.52±303.75 | 440.88±300.58 | 392.27±316.62 | 0.061 |
| **Total choline^a^, Mean±SD** | 298.60±176.44 | 298.66±170.80 | 298.29±202.05 | 0.300 |
| **Vitamin B12^a^, Mean±SD** | 4.35±6.60 | 4.49±7.09 | 3.66±3.38 | 0.143 |
| **Added vitamin B12^a^, Mean±SD** | 0.72±1.98 | 0.76±1.88 | 0.51±2.40 | 0.022 |
| **Vitamin C^a^, Mean±SD** | 76.86±88.95 | 80.09±89.25 | 61.28±86.10 | <0.001 |
| **Vitamin K^a^, Mean±SD** | 100.06±168.41 | 99.07±167.13 | 104.85±175.01 | 0.175 |
| **Calcium^a^, Mean±SD** | 789.61±541.09 | 800.49±554.79 | 737.21±467.80 | 0.245 |
| **Phosphorus^a^, Mean±SD** | 1,141.06±585.95 | 1,143.78±583.30 | 1,127.94±600.46 | 0.466 |
| **Magnesium^a^, Mean±SD** | 254.37±128.99 | 256.50±127.98 | 244.10±133.71 | 0.066 |
| **Iron^a^, Mean±SD** | 12.90±7.79 | 13.11±7.88 | 11.91±7.27 | 0.068 |
| **Zinc^a^, Mean±SD** | 9.47±5.78 | 9.57±5.89 | 9.00±5.18 | 0.359 |
| **Copper^a^, Mean±SD** | 1.10±1.12 | 1.13±1.20 | 1.00±0.57 | 0.071 |
| **Sodium^a^, Mean±SD** | 2,938.10±1,571.35 | 2,959.51±1,563.94 | 2,835.04±1,608.26 | 0.222 |
| **Potassium^a^, Mean±SD** | 2,364.74±1,155.72 | 2,384.69±1,143.60 | 2,268.74±1,212.06 | 0.071 |
| **Selenium^a^, Mean±SD** | 94.25±52.19 | 94.74±51.82 | 91.89±54.08 | 0.451 |
| **Caffeine^a^, Mean±SD** | 152.95±223.20 | 145.72±217.56 | 187.79±246.46 | 0.075 |
| **Theobromine^a^, Mean±SD** | 32.69±70.56 | 31.29±66.61 | 39.41±87.11 | 0.959 |
| **Alcohol^a^, Mean±SD** | 4.73±16.74 | 4.72±15.95 | 4.78±20.17 | 0.242 |
| **Moisture^a^, Mean±SD** | 2,500.90±1,453.12 | 2,453.38±1,338.51 | 2,729.64±1,900.61 | 0.330 |
| **Vitamin D (D2+D3)^a^, Mean±SD** | 4.22±4.19 | 4.31±4.23 | 3.79±3.99 | 0.057 |

a: Student t-test, b: Chi-square test,

**Abbreviation:** PIR, family income to poverty ratio; SD: standard deviation; Pre-DM, prediabetes; mellitus; DM diabetes mellitus; NGR, Normal glucose regulation

**Supplementary Table 2** Associations between WQS-derived nutrient indices and risk of comorbid depression and stroke

| **Characteristic** | **Estimate** | **Standard error** | **z value** | **OR (95% CI)** | **P value** |
| --- | --- | --- | --- | --- | --- |
| **WQS-Negative** | -0.1786 | 0.1559 | -1.1453 | 0.84 (0.62, 1.14) | 0.252 |
| **WQS-Positive** | -0.1268 | 0.1069 | -1.1862 | 0.88 (0.71, 1.09) | 0.236 |

**Note:** The WQS-negative index was constructed to capture potentially harmful nutrient mixtures, while the WQS-positive index reflected potentially protective dietary patterns.

**Abbreviation:** WQS,Weighted Quantile Sum; OR, odds ratios; CI, confidence intervals

**Supplementary Table 3** Nutrient weights from the WQS-negative model for comorbid depression and stroke

| **Mixture Name** | **Mean Weight** |
| --- | --- |
| **Alcohol** | 0.392 |
| **Alpha-carotene** | 0.191 |
| **Added vitamin B12** | 0.097 |
| **Vitamin B1** | 0.091 |
| **Lycopene** | 0.046 |
| **Alpha-tocopherol** | 0.041 |
| **Beta-cryptoxanthin** | 0.026 |
| **Beta-carotene** | 0.017 |
| **Vitamin C** | 0.017 |
| **Sodium** | 0.012 |
| **Copper** | 0.011 |
| **Polyunsaturated fatty acids** | 0.009 |
| **Food folate** | 0.008 |
| **Vitamin K** | 0.006 |
| **Vitamin B12** | 0.006 |
| **Caffeine** | 0.005 |
| **Theobromine** | 0.004 |
| **Cholesterol** | 0.003 |
| **Saturated fatty acids** | 0.003 |
| **Protein** | 0.002 |
| **Vitamin B6** | 0.002 |
| **Lutein+zeaxanthin** | 0.001 |
| **Total choline** | 0.001 |
| **Calcium** | 0.001 |
| **Monounsaturated fatty acids** | 0.001 |
| **Selenium** | 0.001 |
| **Moisture** | 0.001 |
| **Vitamin D** | 0.001 |
| **Total Sugar** | 0.001 |
| **Niacin** | 0.001 |
| **Zinc** | 0.001 |
| **Iron** | 0.001 |
| **Potassium** | 0.001 |
| **Folic acid** | 0.000 |
| **Vitamin B2** | 0.000 |
| **Folate(DFE)** | 0.000 |
| **Total folate** | 0.000 |
| **Total Fat** | 0.000 |
| **Carbohydrate** | 0.000 |
| **Vitamin A** | 0.000 |
| **Phosphorus** | 0.000 |
| **Dietary fiber** | 0.000 |
| **Magnesium** | 0.000 |
| **Energy** | 0.000 |
| **Retinol** | 0.000 |
| **Vitamin E** | 0.000 |

**Note:** Mean weights represent the relative contribution of each nutrient to the WQS-negative index, derived from bootstrap sampling. Higher weights indicate a stronger association with increased risk of comorbidity. In the WQS regression algorithm, the weights estimated within each bootstrap iteration are normalized to sum to 1 by design. The values presented in this table represent the mean of these bootstrap-derived weights across all iterations. Because they are averaged rather than taken from a single iteration, their sum may not equal exactly 1.

**Supplementary Table 4** Nutrient weights from the WQS-positive model for comorbid depression and stroke

| **Mixture Name** | **Mean Weight** |
| --- | --- |
| **Theobromine** | 0.206 |
| **Vitamin E** | 0.194 |
| **Caffeine** | 0.122 |
| **Total Sugar** | 0.082 |
| **Alcohol** | 0.077 |
| **Alpha-tocopherol** | 0.066 |
| **Moisture** | 0.037 |
| **Lutein+zeaxanthin** | 0.036 |
| **Retinol** | 0.032 |
| **Cholesterol** | 0.031 |
| **Vitamin A** | 0.018 |
| **Lycopene** | 0.015 |
| **Folic acid** | 0.015 |
| **Added vitamin B12** | 0.013 |
| **Vitamin D** | 0.010 |
| **Saturated fatty acids** | 0.009 |
| **Zinc** | 0.007 |
| **Monounsaturated fatty acids** | 0.007 |
| **Beta-cryptoxanthin** | 0.005 |
| **Vitamin K** | 0.003 |
| **Protein** | 0.002 |
| **Vitamin C** | 0.002 |
| **Beta-carotene** | 0.002 |
| **Total choline** | 0.001 |
| **Vitamin B12** | 0.001 |
| **Calcium** | 0.001 |
| **Sodium** | 0.001 |
| **Niacin** | 0.001 |
| **Phosphorus** | 0.001 |
| **Dietary fiber** | 0.000 |
| **Food folate** | 0.000 |
| **Folate(DFE)** | 0.000 |
| **Polyunsaturated fatty acids** | 0.000 |
| **Total Fat** | 0.000 |
| **Magnesium** | 0.000 |
| **Total folate** | 0.000 |
| **Vitamin B6** | 0.000 |
| **Carbohydrate** | 0.000 |
| **Selenium** | 0.000 |
| **Energy** | 0.000 |
| **Copper** | 0.000 |
| **Alpha-carotene** | 0.000 |
| **Iron** | 0.000 |
| **Vitamin B1** | 0.000 |
| **Potassium** | 0.000 |
| **Vitamin B2** | 0.000 |

**Note:** Mean weights reflect the relative influence of individual nutrients in the WQS-positive index associated with lower risk of comorbid depression and stroke. In the WQS regression algorithm, the weights estimated within each bootstrap iteration are normalized to sum to 1 by design. The values presented in this table represent the mean of these bootstrap-derived weights across all iterations. Because they are averaged rather than taken from a single iteration, their sum may not equal exactly 1.

**Abbreviation:** WQS,Weighted Quantile Sum
