# Supplementary material for: Using SHAP and LIME to Explain Machine Learning Models Predicting Comorbid Depression and Stroke From Daily Dietary Nutrient Intake in a US Population‐Based Study
Source: Food Sci Nutr. 2025 Dec 30;14(1):e71401. doi: 10.1002/fsn3.71401 (PMC12753580; doi:10.1002/fsn3.71401)
Supplement: Supplementary file 2 — Figure S1: Distribution of class balance before and after SMOTE. Figure S2: Boruta feature importance plot for comorbidity prediction. Figure S3: Accuracy scores of six machine learning models applied to the dataset. Random Forest and LightGBM demonstrated the highest classification accuracy across all models. Figure S4: Fβ scores (β = 1) for each classifier, reflecting the harmonic mean of precision and recall Ensemble models outperformed others, with Random Forest achieving the highest Fβ score. Figure S5: Sensitivity (recall) rates across models, indicating the ability to correctly identify individuals at high comorbidity of depression and stroke risk. LightGBM and Random Forest showed the highest sensitivity. Figure S6: Specificity values of the classifiers, measuring true negative rates. SVM showed the highest specificity, whereas ensemble models maintained balanced sensitivity and specificity. Figure S7: SHAP feature importance plot ranking predictors by their average absolute SHAP values. Features such as age, vitamin B1, and diabetes status contributed most to model predictions. Figure S8: SHAP waterfall plot under covariate‐adjusted model for individual‐level prediction. Figure S9: SHAP dependence plots under covariate‐adjusted model. Figure S10: SHAP waterfall plot under unadjusted model for individual‐level prediction. Figure S11: SHAP dependence plots under unadjusted model. Figure S12: LIME explanation summary for covariate‐adjusted model (representative individual). Figure S13: LIME‐derived feature contributions under covariate‐adjusted model (representative individual). Figure S14: LIME‐derived local explanation summary under unadjusted model (representative individual). Figure S15: LIME‐derived feature contributions under unadjusted model (representative individual). [file FSN3-14-e71401-s002.docx]

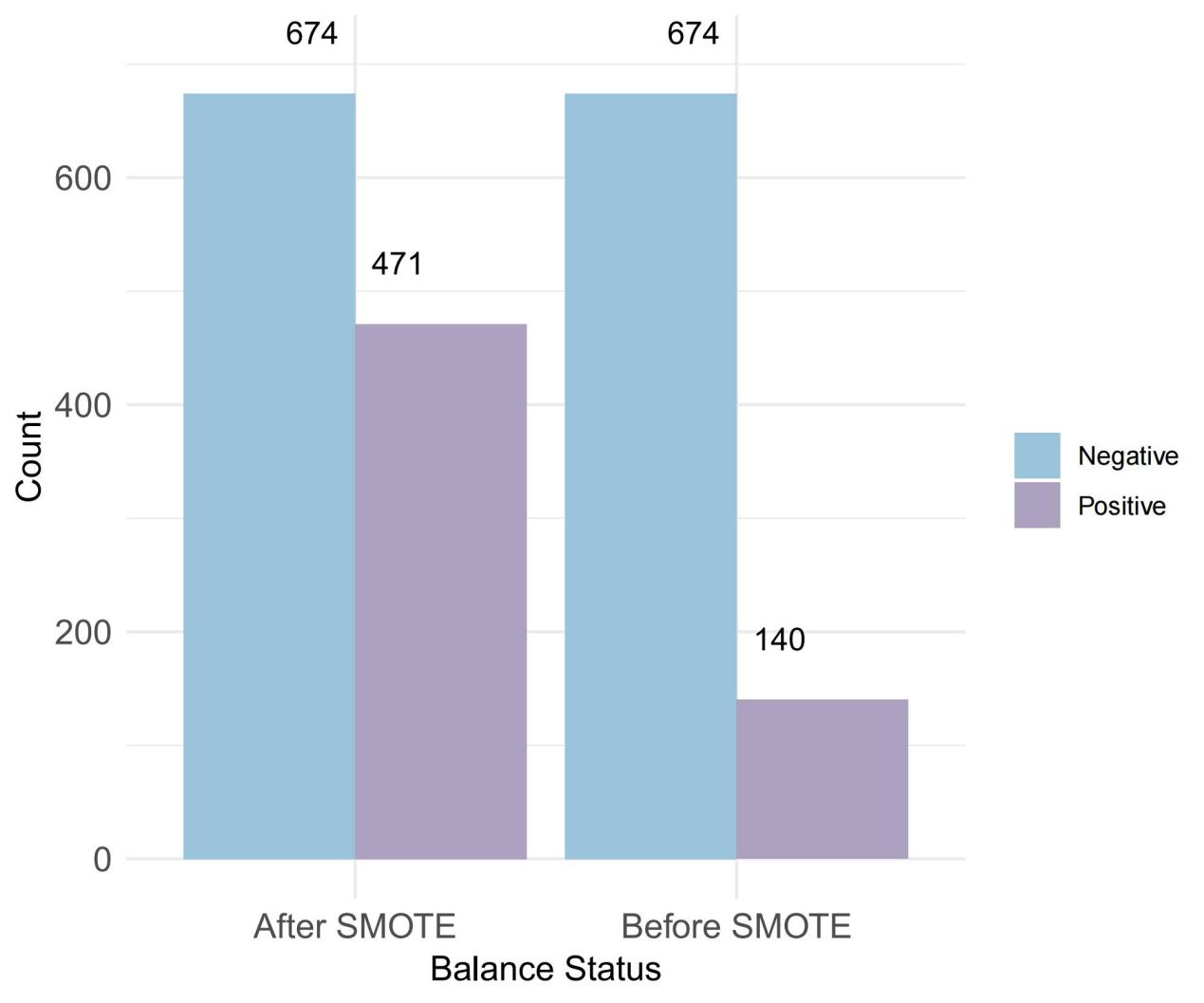


**Supplementary Figure 1** Distribution of class balance before and after SMOTE

**Note:** SMOTE oversampling effectively balanced the number of comorbid (positive) and non-comorbid (negative) samples in the training set

**Abbreviation:** SMOTE, synthetic minority over-sampling technique


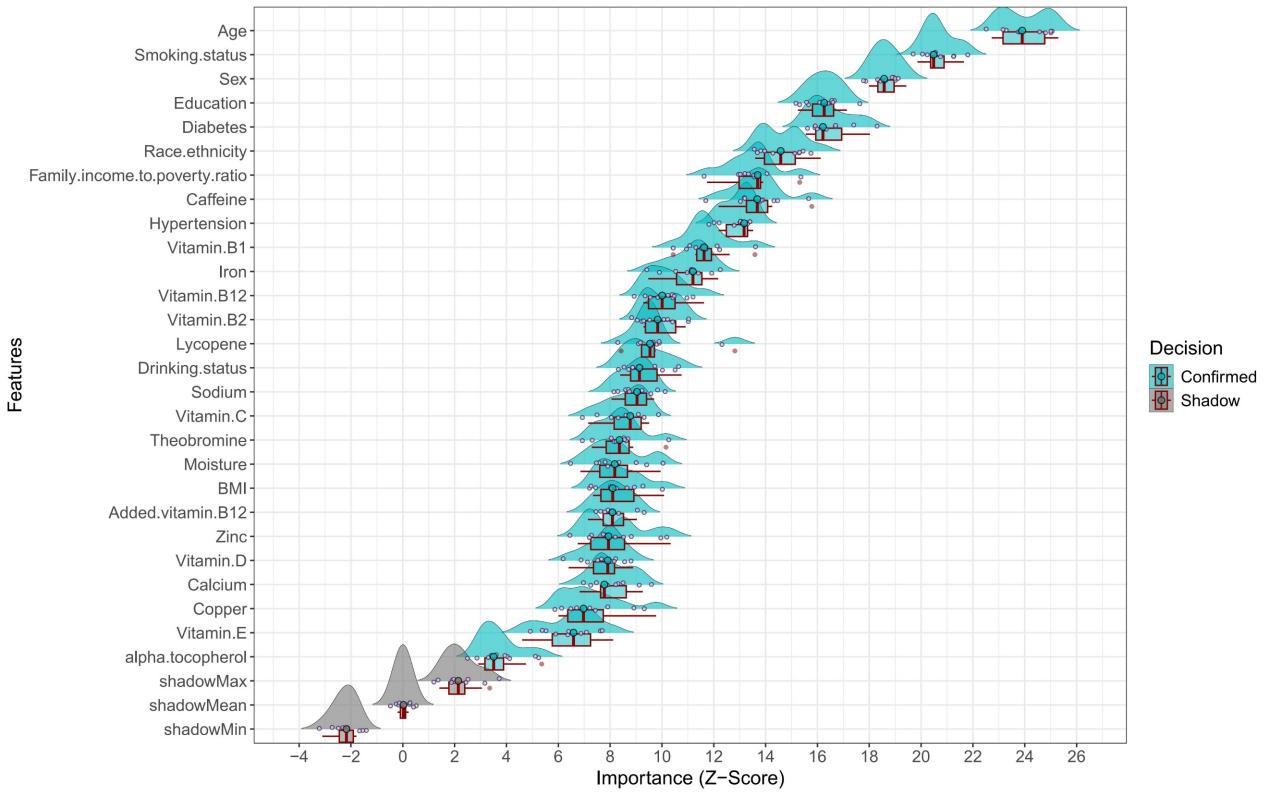


**Supplementary** **Figure 2** Boruta feature importance plot for comorbidity prediction

Features confirmed as important are shown in orange, with importance values based on Z-score comparisons against shadow features (in blue). Nutritional components such as alpha-tocopherol, vitamin E, and caffeine ranked highest


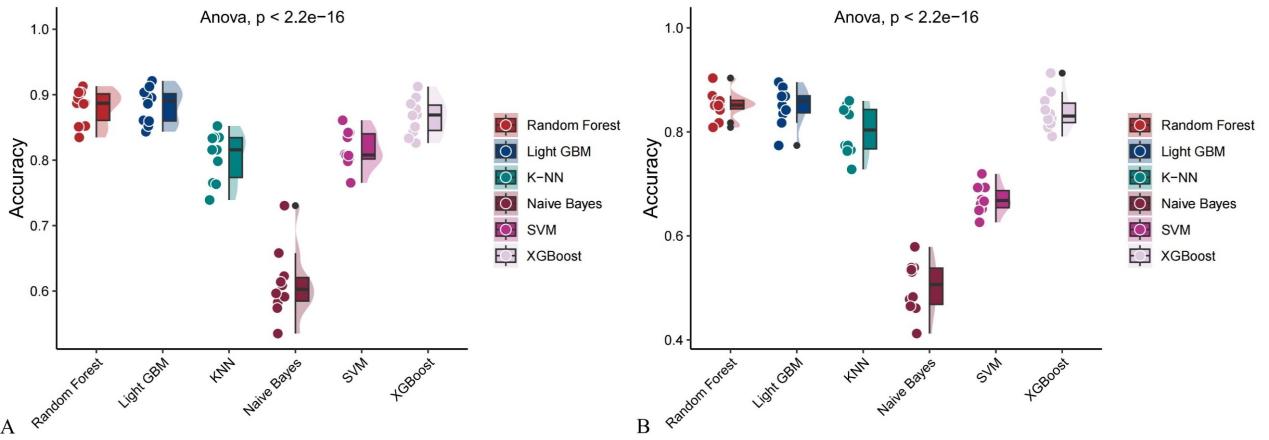


**Supplementary Figure 3** Accuracy scores of six machine learning models applied to the dataset. Random Forest and LightGBM demonstrated the highest classification accuracy across all models

**Abbreviation：**XGBoost, eXtreme Gradient Boosting; SVM, Support Vector Machine; k-NN, k-Nearest Neighbors; LightGBM, Light Gradient Boosting Machine


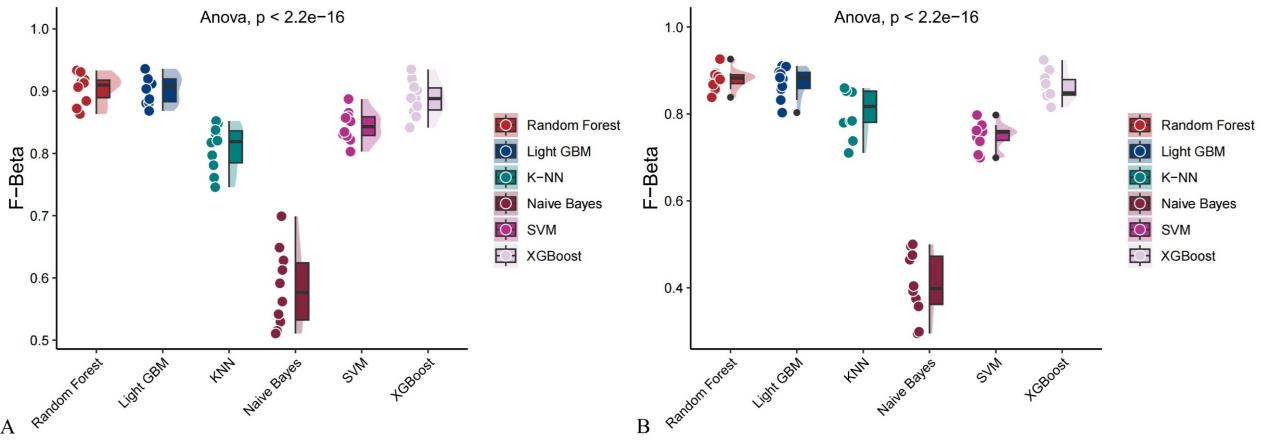


**Supplementary Figure 4** Fβ scores (β=1) for each classifier, reflecting the harmonic mean of precision and recall Ensemble models outperformed others, with Random Forest achieving the highest Fβ score

**Abbreviation：**XGBoost, eXtreme Gradient Boosting; SVM, Support Vector Machine; k-NN, k-Nearest Neighbors; LightGBM, Light Gradient Boosting Machine


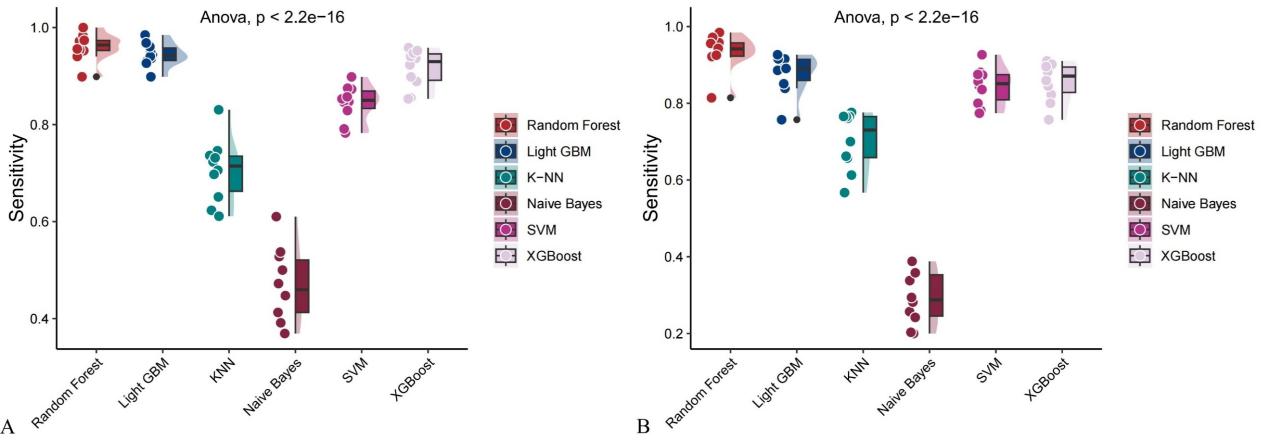


**Supplementary Figure 5** Sensitivity (recall) rates across models, indicating the ability to correctly identify individuals at high comorbidity of depression and stroke risk. LightGBM and Random Forest showed the highest sensitivity

**Abbreviation：**XGBoost, eXtreme Gradient Boosting; SVM, Support Vector Machine; k-NN, k-Nearest Neighbors; LightGBM, Light Gradient Boosting Machine


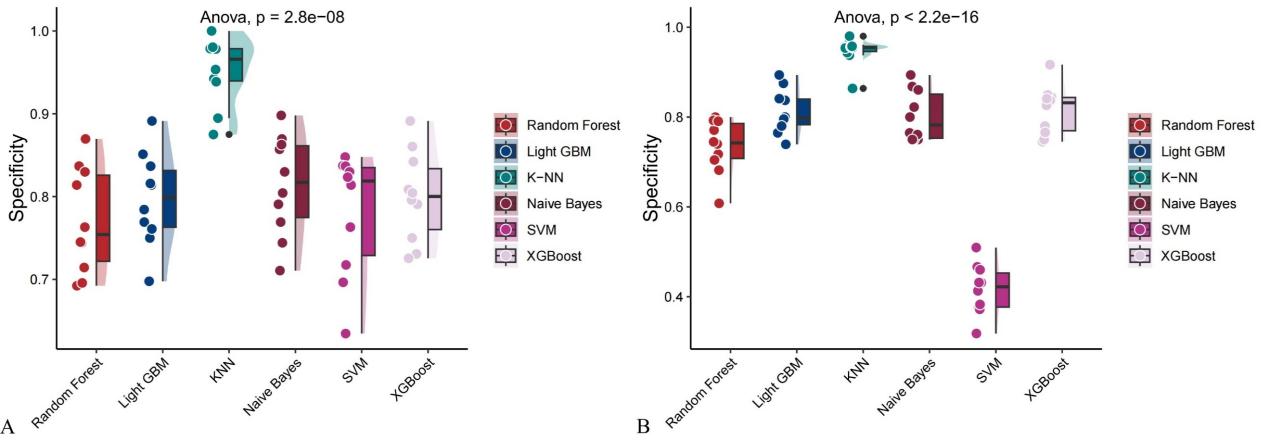


**Supplementary Figure 6** Specificity values of the classifiers, measuring true negative rates. SVM showed the highest specificity, whereas ensemble models maintained balanced sensitivity and specificity

**Abbreviation：**XGBoost, eXtreme Gradient Boosting; SVM, Support Vector Machine; k-NN, k-Nearest Neighbors; LightGBM, Light Gradient Boosting Machine


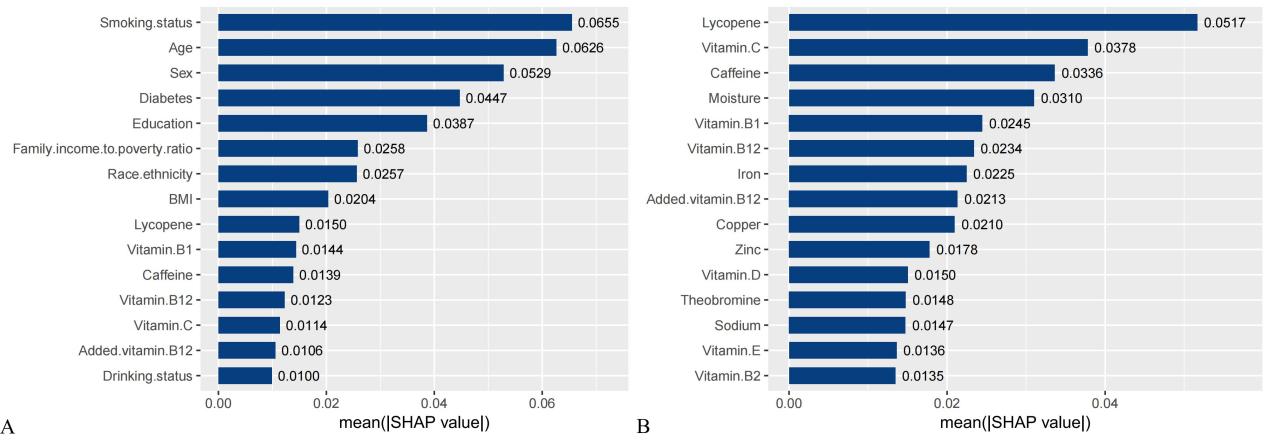


**Supplementary Figure 7** SHAP feature importance plot ranking predictors by their average absolute SHAP values. Features such as age, vitamin B1, and diabetes status contributed most to model predictions

**Abbreviation：**SHAP, SHapley Additive exPlanations


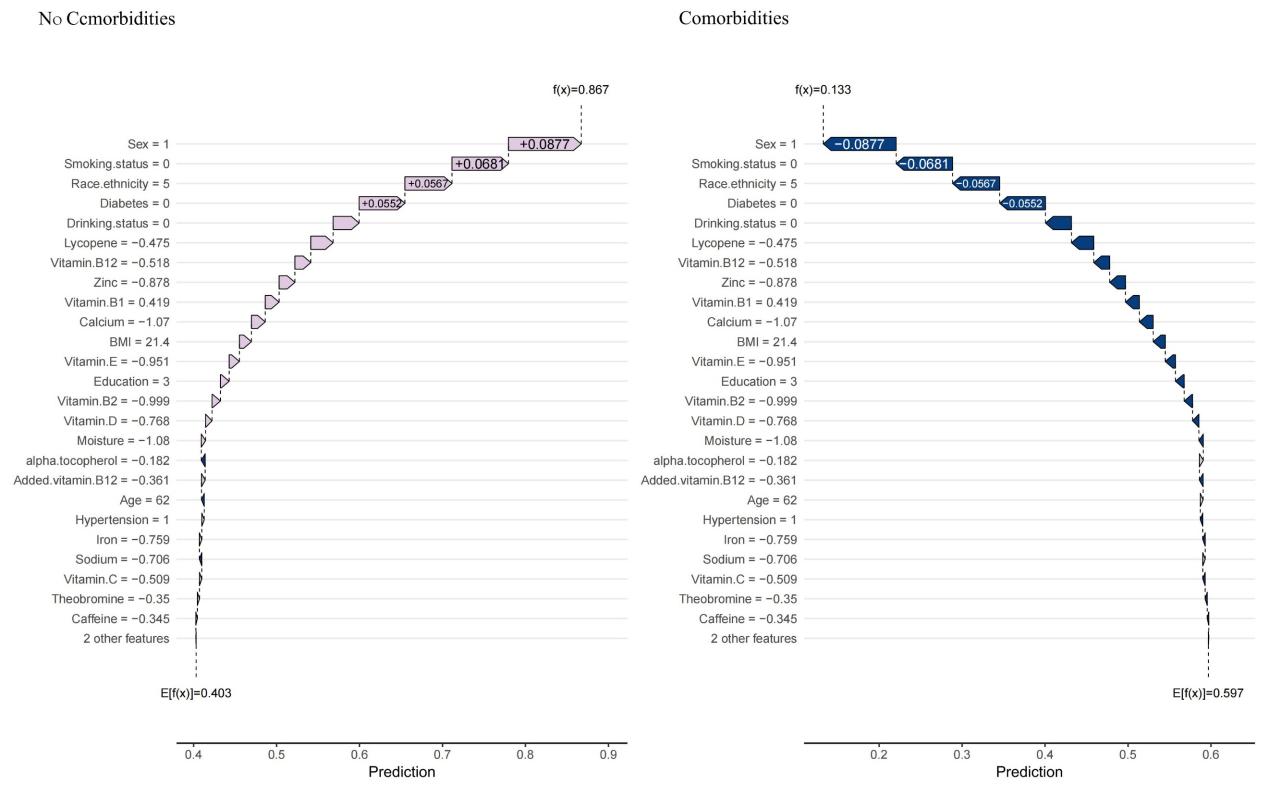


**Supplementary Figure 8** SHAP waterfall plot under covariate-adjusted model for individual-level prediction

**Note:** The left panel presents the prediction for no comorbidities (f(x)=0.867; E[f(x)]=0.403), where all SHAP values contribute positively to elevate the prediction score. The right panel displays the prediction for comorbidities (f(x)=0.133; E[f(x)]=0.597), where all feature contributions are negative, driving the prediction below the expected value

**Abbreviation：**SHAP, SHapley Additive exPlanations


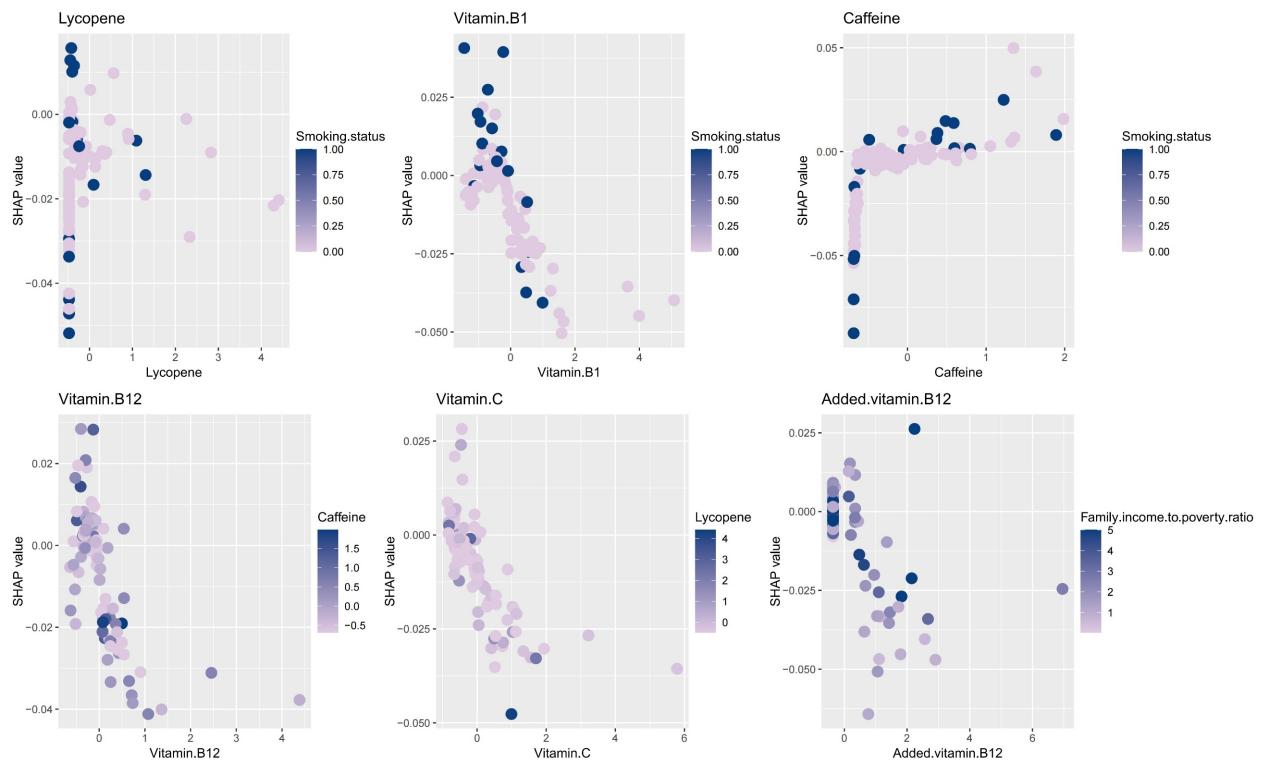


**Supplementary Figure 9** SHAP dependence plots under covariate-adjusted model

**Note:** Dependence plots display the marginal effects of individual features on model output in the covariate-adjusted setting

**Abbreviation：**SHAP, SHapley Additive exPlanations


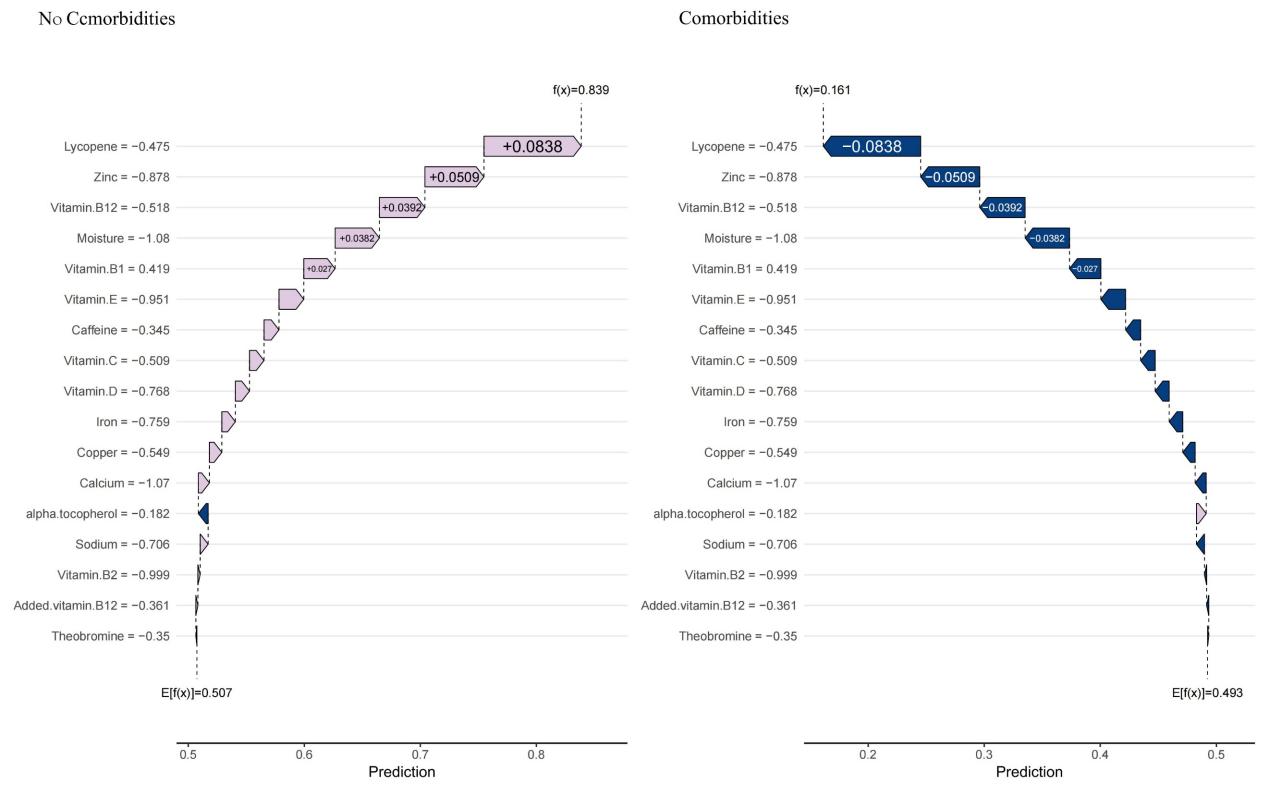


**Supplementary Figure 10** SHAP waterfall plot under unadjusted model for individual-level prediction

**Note:** The left panel presents the prediction for no comorbidities (f(x)=0.839; E[f(x)]=0.507), where all SHAP values contribute positively to increase the predicted probability. The right panel shows the prediction for comorbidities (f(x)=0.161; E[f(x)]=0.493), where all features exert negative contributions.

**Abbreviation：**SHAP, SHapley Additive exPlanations


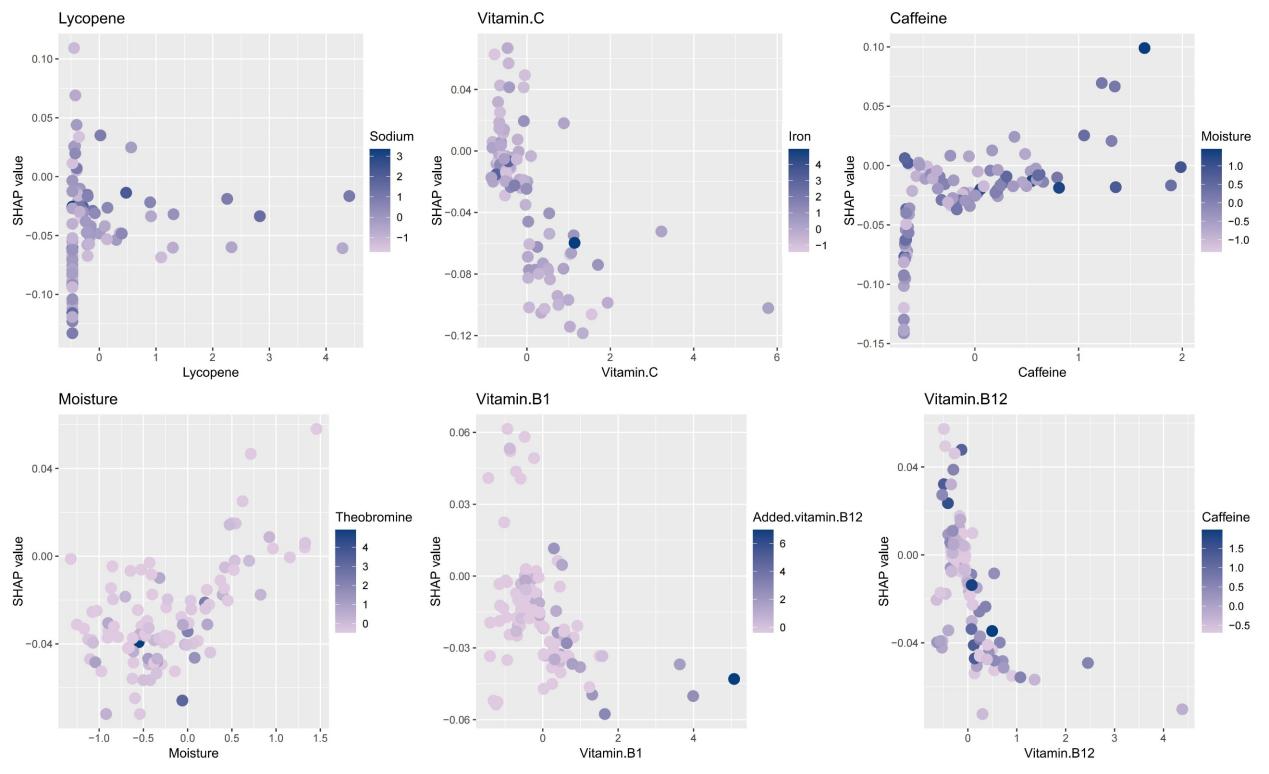


**Supplementary Figure 11** SHAP dependence plots under unadjusted model

**Note:** These dependence plots illustrate the marginal effects of key nutritional features on model predictions for comorbid depression and stroke in the unadjusted setting

**Abbreviation：**SHAP, SHapley Additive exPlanations


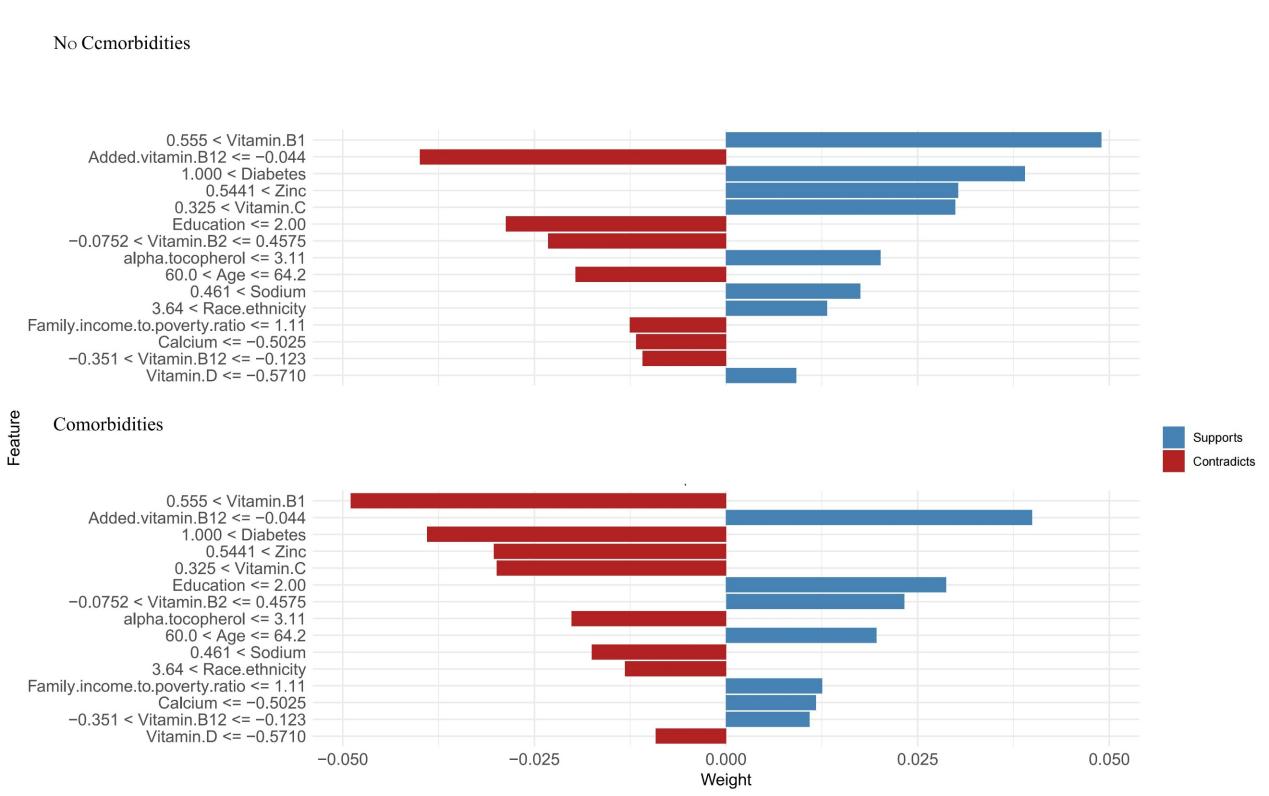


**Supplementary Figure 12** LIME explanation summary for covariate-adjusted model (representative individual)

**Note:** The model assigned a 76% probability of no comorbidities and a 24% probability of comorbidities for the same individual. Feature contributions are displayed for both predicted outcomes, showing how variables support or oppose each classification

**Abbreviation：**LIME, Local Interpretable Model-agnostic Explanations


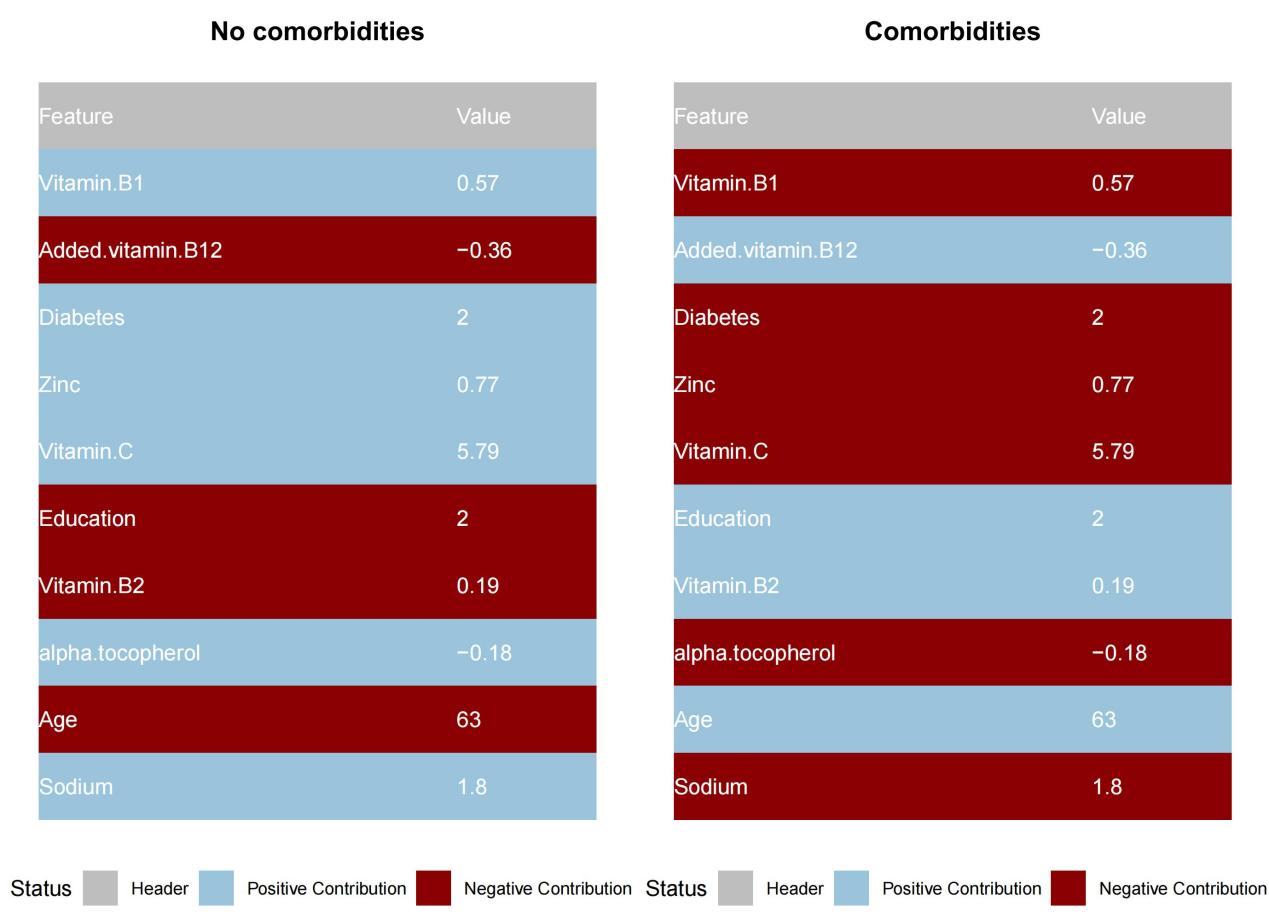


**Supplementary Figure 13** LIME-derived feature contributions under covariate-adjusted model (representative individual)

**Note:** This visualization displays the direction and magnitude of feature contributions for both predicted classes (“no comorbidities” and “comorbidities”) under the covariate-adjusted model using the LIME method. For the “no comorbidities” classification, features such as higher vitamin B1, zinc, and vitamin C levels contributed positively, while lower added vitamin B12, reduced education, and lower alpha-tocopherol exerted negative influence. The direction of contribution for each feature reversed in the “comorbidities” classification

**Abbreviation：**LIME, Local Interpretable Model-agnostic Explanations


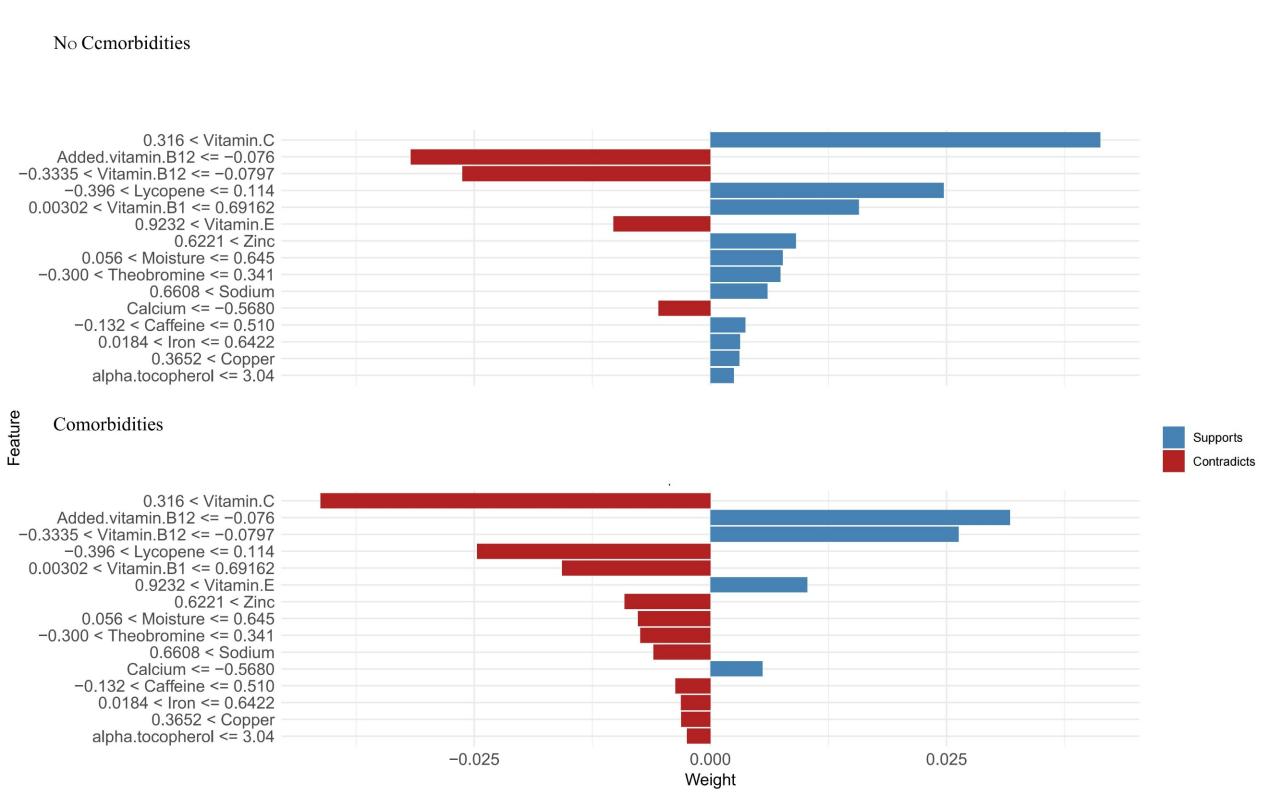


**Supplementary Figure 14** LIME-derived local explanation summary under unadjusted model (representative individual).

**Note:** This plot presents a side-by-side comparison of local explanations for two predicted classes: “comorbidities” (21%) and “no comorbidities” (79%), as generated by the LIME method without adjusting for covariates. It illustrates how individual features contribute to or counteract each classification

**Abbreviation：**LIME, Local Interpretable Model-agnostic Explanations


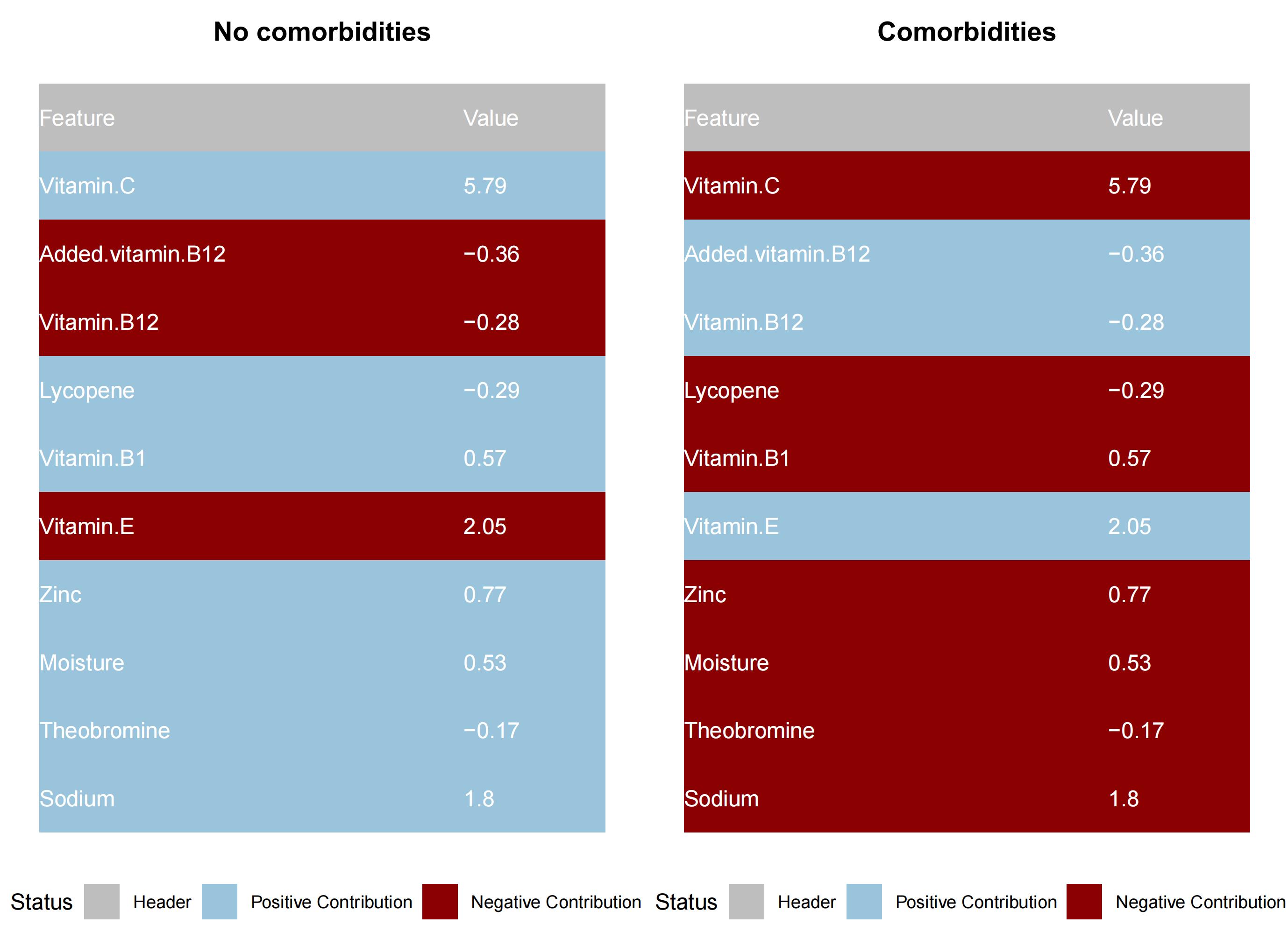


**Supplementary Figure 15** LIME-derived feature contributions under unadjusted model (representative individual)

**Note:** This figure illustrates the directional influence of the top ten nutritional features on the prediction outcomes using the LIME method without covariate adjustment. For the classification of "no comorbidities," features such as higher levels of vitamin C (5.79), vitamin B1 (0.57), zinc (0.77), moisture (0.53), and sodium (1.80) positively contributed to the prediction, while lower values of added vitamin B12 (-0.36), vitamin B12 (-0.28), lycopene (-0.29), vitamin E (2.05), and theobromine (-0.17) contributed negatively. These same features exhibited reverse influences when predicting "comorbidities,"

**Abbreviation：**LIME, Local Interpretable Model-agnostic Explanations
